# RNF216 as a Promising Biomarker for Prognosis, Immunotherapy, and Chemotherapy in LIHC: A Comprehensive Pan-Cancer Analysis and Experimental Validation

**DOI:** 10.7150/jca.125407

**Published:** 2026-01-30

**Authors:** Ke Du, Xiao Liang, Bowen Qin, Zitao Liu, Nanbin Liu, Pincheng Li, Qian Wang, Yajie Qi, Enze Shi, Kun Li

**Affiliations:** National and Local Joint Engineering Research Center of Biodiagnosis and Biotherapy, The Second Affiliated Hospital of Xi'an Jiaotong University, Xi'an, China.

**Keywords:** LIHC, RNF216, prognosis, chemotherapy, immunotherapy

## Abstract

**Background:**

RNF216 belongs to the E3 ubiquitin ligase family and plays a role in the development of various diseases. However, its systematic role in pan-cancer development has not been systematically explored.

**Methods:**

Publicly available data from The Cancer Genome Atlas (TCGA), Gene Expression Omnibus (GEO), and the Human Protein Atlas (HPA) were utilized. The analysis was conducted using R software and online platforms such as STRING, TISIDB, and TISCH to evaluate the role of RNF216. CCK-8 and other experiments have confirmed the function of RNF216 in liver hepatocellular carcinoma (LIHC).

**Results:**

RNF216 was significantly elevated in most tumor tissues relative to adjacent normal tissues; meanwhile, the mutation rate of RNF216 in LIHC tissues was also significantly elevated relative to normal tissues. The expression levels of RNF216 vary in tumor mutational burden (TMB) and microsatellite instability (MSI) across different tumors. It demonstrated significant diagnostic and prognostic value and was associated with clinicopathologic features in multiple cancers, especially in LIHC. The protein-protein interaction (PPI) network and GSCALite suggested that RNF216 and its co-expressed genes may promote tumor growth by regulating mitosis, cell death, and DNA damage. Gene Set Enrichment Analysis (GSEA) further revealed a positive correlation between RNF216 and cell cycle regulation pathways. RNF216 is significantly related to immune cell infiltration and expressed in various types of immune cells. Knockout of RNF216 mediated by siRNA inhibits cell proliferation and reduces the migration and invasion capability of HepG2 and Hep3B cells.

**Conclusion:**

RNF216 is strongly upregulated in various tumors, including LIHC, and plays an extremely important role in tumor diagnosis and prognosis. Targeted knockout of RNF216 is beneficial for improving the efficacy of immunotherapy and chemotherapy.

## Introduction

Cancer is one of the leading causes of disease and death worldwide, posing a severe threat to public health by increasing clinical burdens, medical costs, and negatively impacting health and lifespan. In East Asia, cancer has become a major public health issue, with an estimated 304,754 new cancer cases and 84,019 cancer deaths in South Korea by 2025 1. In 2022, there were approximately 482,4700 new cancer cases and 2574200 new cancer deaths in China. Lung cancer, colorectal cancer, thyroid cancer, liver cancer, and stomach cancer are the top five types of cancer 2. Data from the United States shows that it was estimated that nearly 500 people would die of cancer every day in 2025, most of whom will die of lung cancer, about 124,730 cases. The number of deaths caused by lung cancer is almost 2.5 times that of the second largest colorectal cancer and the third largest pancreatic cancer 3. The most common histological subtype of non-small cell lung cancer (NSCLC) is lung adenocarcinoma (LUAD), originating from the bronchial mucosal epithelium and sometimes derived from malignant transformation of the larger bronchial mucinous glands 4. Despite significant progress in clinical treatments of LUAD, including immunotherapy, chemotherapy, radiotherapy, and surgery, the 5-year survival rate of NSCLC remains stable at a low level of 26% 5. Liver cancer is the third leading cause of cancer-related deaths and the sixth most common cancer worldwide 6. LIHC is the leading cause of death in liver cirrhosis cases, accounting for over 80% of primary liver cancer patients 7. Current LIHC treatments include surgical procedures, local-regional therapies, and systemic treatments. Surgical resection and liver transplantation are the preferred treatment options for early LIHC. Localized treatment, such as radiofrequency ablation (RFA) and transarterial chemoembolization (TACE), are used for patients with intermediate-stage or inoperable tumors, while systemic therapy is mainly reserved for advanced cases 8. However, the long-term outlook for LIHC remains bleak due to high recurrence rates after liver resection—about 80% within five years 9. Patients with early-stage LIHC undergoing curative procedures may survive more than 10 years on average, whereas those with late-stage LIHC usually have a median survival period of only 1 to 3 years 10. In conclusion, discovering novel biomarkers or therapeutic targets for diagnosing and treating LIHC and LUAD is critically important.

Ring finger protein 216 (RNF216, also known as TRIAD3) is a versatile E3 ubiquitin ligase encoded by the RNF216 gene located at 7p22.1. The RNF216 protein consists of approximately 866 amino acids 11. RNF216 interacts with various proteins and receptors, including Toll-like receptors (TLR), tumor necrosis factor receptor-associated factor 3 (TRAF3), etc. RNF216 regulates the expression levels of these proteins through ubiquitination 12. Mutations in the RNF216 can cause damage to the white matter, hippocampus, cerebellum, pituitary gland, and hypothalamus involved in reproductive endocrine regulation 13. In terms of disease connections, loss of function in RNF216 is associated with neurodegenerative disorders such as Gordon-Homes syndrome (GHS) and Huntington-like disease, which feature cerebellar ataxia, hypogonadotropic hypogonadism, and dementia 14. Regarding cancer, in LUAD, RNF216 is a key oncogenic driver that promotes disease progression through p53 ubiquitination and ferroptosis inhibition 11. In ovarian cancer, overexpression of miR-520b boosts cell growth by inhibiting RNF216 15. In gastric cancer, structural analysis identified the TSC2-RNF216 fusion gene, which may impair tumor suppressor pathways and simultaneously activate tumorigenesis 16. RNF216 reduces radiation-induced DNA damage and apoptosis in glioblastoma (GBM) by promoting p53 ubiquitination, thereby contributing to tumor resistance 17. Despite the aforementioned studies, there is still a lack of comprehensive research exploring the relationship between RNF216 and pan-cancer.

Tumor bioinformatics plays an extremely important role in tumor research and precision medicine. Driven by the development of technology, large-scale tumor omics data is gradually increasing 18. In this research, we conducted a comprehensive analysis of the value of RNF216 in pan-cancer, using platforms such as Gene Expression Omnibus (GEO), The Cancer Genome Atlas (TCGA), and Human Protein Atlas (HPA) databases, along with R and various bioinformatics tools. The focus was on examining the association of RNF216 with clinicopathological features, co-expressed genes, protein-protein interactions (PPIs), mutation status, ferroptosis, m6A methylation modifications, immune infiltration, and drug response in LIHC and LUAD. Besides, we conducted additional experiments to confirm some key findings. The targets of this study are to elucidate the value of RNF216 in the diagnosis and prognosis of pan-cancer, providing insights for developing new drugs for pan-cancer therapy.

## Materials and Methods

### Data collection and processing

The HPA database (https://portal.gdc.cancer.gov/) was utilized to assess the RNA level of RNF216 in healthy mankind organs, tissues, and at cell level. Specifically, data were collected on RNF216 RNA expression in immune cells and individual cells, along with RNF216 protein scores. The HPA database also provides subcellular localization of RNF216 by visualizing protein expression in mankind tissues through indirect immunofluorescence and using immunohistochemistry (IHC) labeling 19. Consensus and GTEx data were also employed to evaluate RNF216 expression across various normal tissues.

We obtained RNA sequencing and clinical information data for various tumors from the TCGA database (*https://portal.gdc.cancer.gov/*) 20. All available cancer tissue samples and their corresponding normal tissue samples were included in this study, while samples lacking complete clinicopathologic data were excluded. The 'ggplot2' package in R was utilized to analyze and present the differential expression of RNF216 across the entire tumor sample cohort, pan-cancer cell line samples, normal samples, and matched tumor-normal pairs. The expression of RNF216 across different tumors was further validated using the following datasets from the GEO database: GSE19804 21, GSE37182 22, GSE45267 23, GSE76427 24, and GSE84598 25. These are commonly used datasets for pan-cancer research and bioinformatics analysis, and have significant value in clinical applications and scientific research practices.

### Patients and tissue samples

Three pairs of frozen LIHC tumor tissues and matched non-tumor tissues were collected from the Second Affiliated Hospital of Xi'an Jiaotong University between 2018 and 2020. All tissues came from the sample bank at the National-Local Joint Engineering Research Center for Biological Diagnosis and Biotherapy within the same hospital. All participants signed an informed consent form before participating, and all processes followed ethical guidelines and obtained approval from the Ethics Committee of the Second Affiliated Hospital of Xi'an Jiaotong University.

### Diagnostic and prognostic value of RNF216 in pan-cancer

We examined the relationship between RNF216 expression and clinicopathologic features of pan-cancer in the TCGA database, including age, sex, BMI, tumor stage, histological grade, anatomical site, tumor status, residual tumor, treatment response, and smoking history. We evaluated RNF216 as a potential pan-cancer diagnostic biomarker using receiver operating characteristic (ROC) curves. Visualization was performed using the 'pROC' and 'ggplot2' packages in R. In addition, univariate Cox regression analysis was conducted using the 'survival' and 'ggplot2' packages to determine the associations between RNF216 expression and overall survival (OS), disease-specific survival (DSS), and progression-free interval (PFI) in pan-cancer patients 26. LIHC and LUAD cases were split into high- and low-expression groups based on RNF216 levels. Kaplan-Meier curve analysis was utilized with the 'survival' package for OS, DSS, and PFI in LIHC and LUAD patients grouped by RNF216 expression. Risk factors were analyzed with the 'ggrisk' package, with visualization using the 'ggplot2' package.

### Relationship between RNF216 expression and clinicopathologic characteristics in LIHC and LUAD

To clarify the potential link between RNF216 expression and various clinicopathologic indicators in LIHC and LUAD, we used Wilcoxon or Kruskal-Wallis tests. These indicators included age, BMI, histological grade, pathological T stage, overall stage, tumor status, pathological N stage, alpha-fetoprotein (AFP) levels and others. Additionally, to gain deeper insights into the relationship between RNF216 and specific clinical parameters in LIHC and LUAD, we employed chi-square tests and logistic regression analyses. Variables with statistical significance were visualized using the 'ggplot2' package.

### Mutation status of RNF216 across the pan-cancer study

Based on the cBioPortal database (*https://www.cbioportal.org*), the frequency of genetic alterations in RNF216 was examined across different tumor types 27. Using TCGA data, we explored the correlation between RNF216 expression levels and tumor mutational burden (TMB) and microsatellite instability (MSI) in different tumors. For LIHC and LUAD, we obtained STAR counts data, mutation MAF files, and relevant clinical information from TCGA. In R, we visualized somatic mutation data from LIHC patients with the 'maptools' package. Additionally, we analyzed copy number variations (CNVs) of RNF216 in LIHC by the GSCA database (*https://guolab.wchscu.cn/GSCA/#*), retrieving data from (*https://guolab.wchscu.cn/GSCA/#/mutation*) 28.

### Differentially expressed genes (DEGs) of RNF216 and enrichment analysis in LIHC and LUAD

LIHC and LUAD cases were separated into high- and low-expression groups based on median expression levels of RNF216. Differential expression analysis was performed by the 'limma' package, with visualization through the 'ggplot2' package. DEGs were identified using the following threshold criteria: LIHC: |log2 fold change (FC)| > 2 and adjusted *p*-value (*p*adj.) < 0.05; LUAD: |log2FC| > 1.5 and *p*adj. < 0.05. Then, we utilized the 'clusterProfiler' package for functional enrichment analysis, including Gene Ontology (GO), Kyoto Encyclopedia of Genes and Genomes (KEGG), and Gene Set Enrichment Analysis (GSEA), to understand the biological significance of the DEGs 29. Significantly enriched pathways were identified using combined thresholds of normalized enrichment score (NES), False Discovery Rate (FDR) < 0.25, and *p*adj. < 0.05.

### Analysis of the RNF216-related gene and PPI network in LIHC and LUAD

We separately evaluated the 30 genes with the strongest correlation with RNF216 in LIHC and LUAD, and utilized the 'ggplot2' package for visualization. In addition, we examined the PPI network of RNF216 by the STRING database (*https://cn.string-db.org/*) 30. We picked the top 10 genes with the strongest relationship in the LIHC and LUAD co-expression networks, as well as the top 10 proteins with the highest interaction points in the PPI network. We used the Tumor Immune Evaluation Resource 2.0 (TIMER2) (*http://timer.cistrome.org/*) to determine the expression of RNF216 and its related genes in different cancers 31. The correlation of these genes in LIHC and LUAD was verified, with visualization by the 'circlize' package.

### Prognostic significance analysis of RNF216 co-expressed genes in LIHC and LUAD

We assessed the prognostic importance of co-expressed genes in LIHC and LUAD and analyzed their expression levels across various tumor stages. GSCALite (*https://guolab.wchscu.cn/GSCA*) was also utilized to examine the signaling pathways commonly enriched in LIHC and LUAD by RNF216 and its associated genes with prognostic value 32.

### Correlation between RNF216 expression in pan-cancer patients and the tumor immune microenvironment (TIME)

The connection between RNF216 expression and immune microenvironment infiltration across various malignant tumors was analyzed using the online database TISIDB (*http://cis.hku.hk/TISIDB/index.php*) 33. These regulators include tumor-infiltrating lymphocytes (TILs), immune stimulators, immune suppressors, major histocompatibility complex (MHC), chemokines, and receptors. We use the website's online search and plotting functions to calculate correlation coefficients, generate correlation heat maps, and plot linear regression equations, selecting representative fitting line graphs for display.

### Single-cell sequencing analysis of RNF216 in LIHC and LUAD

We downloaded single-cell data in .h5 format and LIHC annotation results from the Tumor Immune Single-Cell Hub (TISCH) database (*http://tisch.compbio.cn/*) 34. These data were handled and examined by R, MAESTRO and Seurat, with cell clustering and subgroup identification performed through the t-distributed stochastic neighbor embedding (t-SNE) method.

### Immunotherapy and chemotherapy response analysis of RNF216 in LIHC

We collected RNA sequencing expression profiles and related clinical data of LIHC from the TCGA dataset. Then, the Tumor Immune Dysfunction and Exclusion (TIDE) algorithm was utilized to forecast potential responses to immune checkpoint inhibitor (ICI) therapy. Additionally, the Genomic Drug Sensitivity in Cancer (GDSC) and Cancer Therapy Response Portal (CTRP) databases were employed to predict chemotherapy responses in LIHC cases with high- and low-RNF216 expression 35. Drug sensitivity predictions were carried out with the 'prophetic' package, estimating half-maximal inhibitory concentration (IC50) values through ridge regression analysis 36.

### Correlation analysis of RNF216 with ferroptosis and m6A methylation-related genes in LIHC and LUAD

Ferroptosis indicates the disorder of lipid oxidation metabolism within cells, leading to the formation of toxic lipids and resulting in cell death. m6A methylation is an RNA methylation marker, particularly the methylation of the sixth nitrogen atom on adenine (A) in RNA, which affects transcription and translation processes. We evaluated the relationship between RNF216 and genes related to ferroptosis in LIHC and LUAD, as well as m6A-related genes. Ferroptosis-related genes were obtained from a systematic analysis of ferroptosis in cancer by Ze-Xian Liu *et al.* 37. m6A methylation-related genes were identified from Li-Lan Yi *et al.*'s study on m6A methylation regulators in 769 cases of head and neck squamous cell carcinoma (HNSC) 38.

### Cell culture and siRNA transfection

Human LIHC cell lines, HepG2, Hep3B, and the normal human hepatocyte cell line LO2 were obtained from the American Type Culture Collection (ATCC, USA), MHCC97H was obtained from the China Center for Type Culture Collection (China). All cell lines were authenticated by their respective sources and were used for experiments within 5 to 10 passages to ensure genetic stability and phenotypic consistency. The cell lines were cultured in DMEM medium (Corning, USA), added with 10% FBS (Corning, USA), 1% penicillin-streptomycin antibiotic. All cells were cultivated in a humidified incubator at 37 °C with 5% CO2. The siRNAs targeting RNF216 (siRNF216#1; siRNF216#2) and the negative control (si#NC) were devised and composed by Tsingke (Beijing, China). The HepG2 and Hep3B cells were transfected with siRNA by Lipofectamine 3000 Reagent (Invitrogen, Carlsbad, CA, USA) based on the manufacturer's instructions. After 48 h, all cells were harvested for subsequent RNA and protein extraction. The related siRNA sequences are as follows:

si#NC forward: (5′-UUCUCCGAACGUGUCACGUTT-3′), si#NC reverse: (5′-ACGUGACACGUUCGGAGAATT-3′); siRNF216#1 forward: (5′-GAGACAGAGCUGUUAUCAATT-3′), siRNF216#1 reverse: (5′-UUGAUAACAGCUCUGUCUCTT-3′); siRNF216#2 forward: (5′-GACUCUGGAAAGAACAUAATT-3′), siRNF216#2 reverse:(5′-UUAUGUUCUUUCCAGAGUCTT-3′).

### Quantitative real-time PCR and western blot analysis

Total RNAs were extracted using an RNAiso Plus Kit (Takara, Japan) and reverse-transcribed using a PrimeScript™ RT Master Mix Kit (Takara, Japan). Quantitative real-time PCR analysis (qRT-PCR) was managed using CFX96TM Real-time PCR system (Bio-Rad, CA, USA) and a TB Green kit (Takara, Japan) to determine the relative expression of target genes. Gene expression data were analyzed via the 2⁻ΔΔCt method; GAPDH acted as the internal control for normalization. The primer sequences utilized in this research are listed below.

RNF216 forward: (5′-GGCGACTTGAGTGACGATTC3′), RNF216 reverse: (5′-CTCAGAATCATCTTCTGTCAGGAT-3′); GAPDH forward: (5′-GTCTCCTCTGACTTCAACAGCG-3′), GAPDH reverse: (5′-ACCACCCTGTTGCTGTAGCCAA-3′).

Proteins from tissues and cells were extracted in radioimmunoprecipitation assay (RIPA) lysis buffer (P0013, Beyotime, China) with protease and phosphatase inhibitors (CWBIO, China). The same amounts of proteins were electrophoresed by 8-12% SDS-PAGE, transferred onto 0.2 μm PVDF membranes, and incubated with primary antibody of RNF216 (1:2000, Proteintech, USA) and GAPDH (1:5000, Proteintech, USA) at 4 °C overnight, followed by incubation with appropriate HRP-conjugated secondary antibody (1:2000, Cell Signaling Technology, USA) at normal atmospheric temperature for 1 h. Signals were detected by Immobilon ECL substrate (Bio-Rad, USA).

### Cell counting kit (CCK)-8 and EdU assays

Cell proliferative capability was determined by the CCK-8 kit (Beyotime, Shanghai, China) and EdU Kit (BeyoClick™ EdU-488, Beyotime, Shanghai, China) according to the manufacturer's manual. In CCK-8 assays, the cells were suspended in 100 μL DMEM medium and seeded in a 96-well plate at a density of 1 × 10^4^ cells per well. The culture medium was added with 10 μL CCK-8 reagents at different times and then incubated for 1 h. The cell proliferation was determined by detecting the optical absorbance density at 450 nm. EdU assays were performed within 24 h after transfection experiments. In EdU assays, 5 × 10^3^ cells were incubated with EdU and then fixed with 4% PFA fixation solution. The cells were stained with Click reaction buffer for 30 min and then incubated with Hoechst 33342 for 30 min. Visualization was conducted under a fluorescence microscope (Leica, Germany). The EdU positive cells in five random regions (> 60 cells in each region) in each of three independent assays were quantified using Image J.

### Cell migration and invasion assays

Migration or Matrigel invasion assays were implemented using 24-well transwell inserts equipped with an 8-μm pore size membrane (Corning, USA). The cells (5 × 10^4^ cells) were inoculated into the upper chamber with 200 μL serum-free medium. The lower compartment contained 500 μL complete medium. After 24 h, the upper chambers were removed, and the cells on the upper side of the membrane were completely removed through gentle swabbing. The migrated or invaded cells were fixed with 4% PFA for 15 min and stained with 0.5% crystal violet for 10 min. The migrated cells in each well were tallied under a phase contrast microscope, with at least 5 fields of view captured per chamber. Quantitative analysis was conducted using ImageJ.

### Statistical analysis

All statistical analyses were performed using R (version 4.5.1) and GraphPad Prism (version 8.0). The normality of data distribution was assessed using the Shapiro-Wilk test. All experiments were independently replicated three times, and data are expressed as the mean ± standard deviation (SD). Comparisons between two groups were performed using Student's *t*-test and the Wilcoxon signed-rank test for normally and non-normally distributed data, respectively. For comparisons among three or more groups, one-way ANOVA with Dunnett's post-hoc test was used for parametric data, while the Kruskal-Wallis test with Bonferroni correction was employed for nonparametric data. Correlations were analyzed using Spearman's rank correlation coefficient. *P <* 0.05 was considered statistically significant. Significance levels are denoted as follows: *P < 0.05, **P < 0.01, ***P < 0.001, and ****P < 0.0001; ns (not significant) indicates *P* ≥ 0.05.

## Results

### Expression of RNF216 in normal human organs and tissues

RNF216 mRNA is widely expressed in many human organs and tissues (Fig. S1A). Consensus dataset analysis shows that RNF216 is mainly found in the testes, colon, endometrium, myocardium, skeletal muscle, spleen, small intestine, lymph nodes, ovaries, and bladder (Fig. S1B). The GTEx dataset analysis indicates that RNF216 is primarily present in the testes, colon, endometrium, myocardium, skeletal muscle, spleen, small intestine, ovaries, bladder, and fallopian tubes (Fig. S1C). Additionally, data from the HPA dataset reveal that RNF216 is mostly expressed in cardiac and skeletal muscle, lymph nodes, bone marrow, testes, pancreas, smooth muscle, spleen, parathyroid glands, and seminal vesicles (Fig. S1D). Regarding protein expression, the RNF216 protein is detected in the cerebral cortex, hippocampus, thyroid, parathyroid, adrenal gland, bronchus, lung, stomach, duodenum, small intestine, liver, gallbladder, kidney, bladder, testes, ovaries, fallopian tubes, endometrium, cervix, placenta, mammary glands, smooth muscle, soft tissue, adipose tissue, appendix, spleen, lymph nodes, tonsils, and bone marrow (Fig. S1E). RNF216 was mainly expressed in eosinophils, neutrophils, NK cells, naive CD8 T cells, and naive CD4 T cells, with relatively low immune cell specificity (Fig. S1F). As illustrated in Fig. S1G, RNF216 gene expression varies significantly across tissues and cell types, with especially high levels in germ cells of the testes and ovaries—particularly in late spermatocytes, spermatogonia, and early spermatocytes. Elevated expression was also observed in neural and muscle cells of the brain and eye, as well as in hematopoietic and immune cells from multiple organs. Subcellular location and protein structure of RNF216 were obtained from the HPA database (Fig. S1H-J). Immunofluorescence studies in A-431, U-2OS, and U-251MG cells revealed RNF216's subcellular distribution, with green indicating the location and intensity of its expression. The figures presented that RNF216 is mainly positioned in the cytoplasm and nucleoplasm. These three cell lines play vital roles in cancer studies and are extensively used in cell and molecular biology research, as they are relatively convenient to cultivate in laboratory settings, ensuring the accuracy and reproducibility of experimental results.

### RNF216 expression is significantly elevated in various tumors, including LIHC

**Figure 1:**
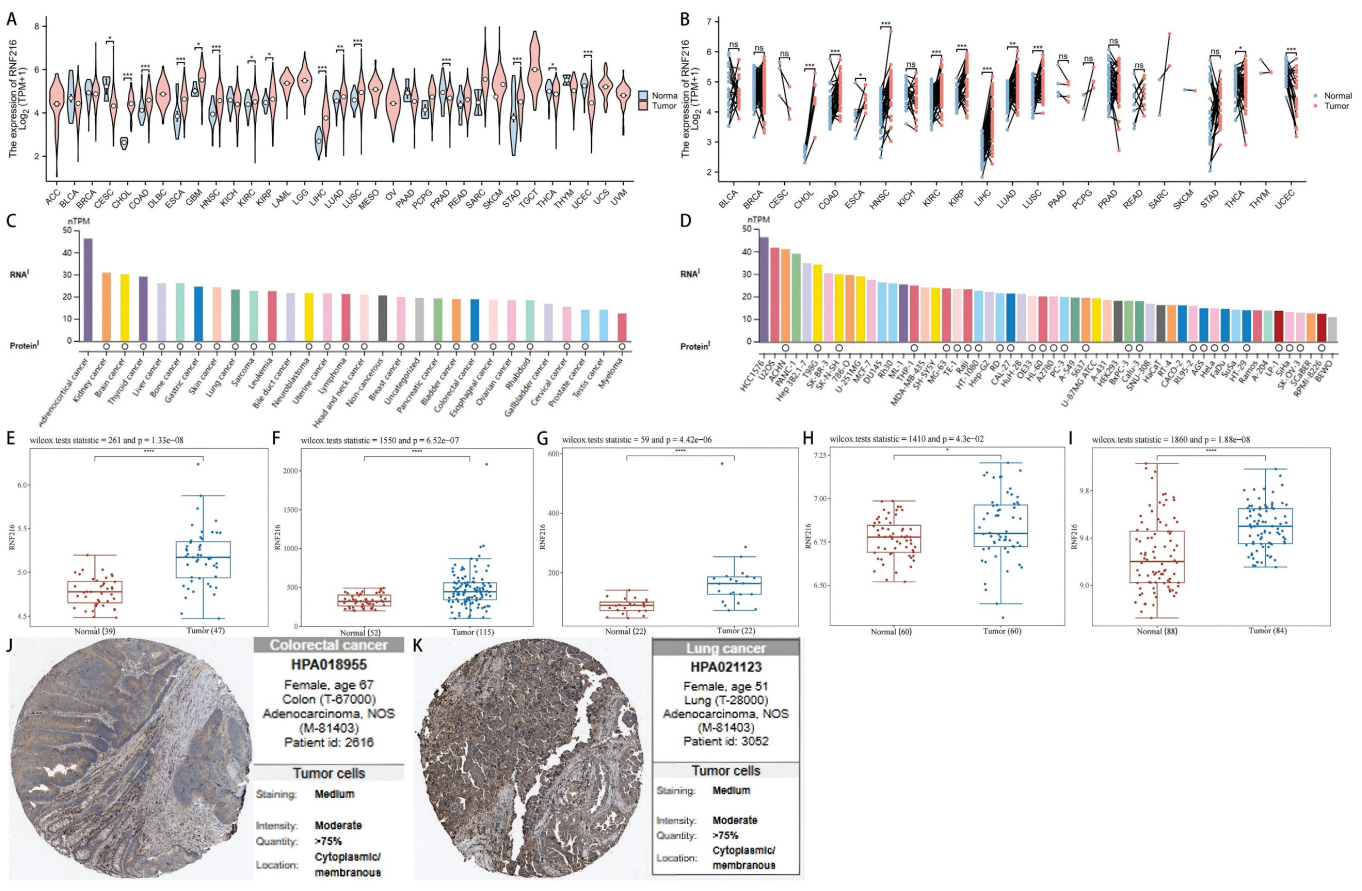
**RNF216 expression landscape.** (A, B) TCGA database showing RNF216 expression levels in unpaired and paired pan-cancerous tissues. (C) The mRNA expression of RNF216 across different types of cancer. (D) The mRNA expression of RNF216 in various pan-cancer cell lines. (E-G) The mRNA expression of RNF216 in LIHC based on data from the GEO datasets GSE19804, GSE37182, GSE45267, GSE76427, and GSE84598. (H) The mRNA expression of RNF216 in LUAD from the GEO database. (I) The mRNA expression of RNF216 in COAD from the GEO database. (J, K) The protein expression of RNF216 in colorectal and lung cancers by IHC staining from the HPA database. (*P < 0.05; **P < 0.01; ***P < 0.001).

After analyzing pan-cancer data from TCGA, we observed differential expression of RNF216 across 15 unpaired cancer types and 11 paired cancer types. These include cholangiocarcinoma (CHOL), colorectal adenocarcinoma (COAD), esophageal carcinoma (ESCA), HNSC, renal clear cell carcinoma (KIRC), renal cell carcinoma (KIRP), LIHC, LUAD, and lung squamous cell carcinoma (LUSC). RNF216 was upregulated in these cancer types, while it was downregulated in thyroid carcinoma (THCA) and uterine endometrial carcinoma (UCEC) (Fig. 1A, B). Additionally, data of the HPA dataset indicated that RNF216 mRNA was mainly distributed in adrenal cortical carcinoma, renal cell carcinoma, and brain cancer cell lines, whereas RNF216 protein was primarily present in renal cell carcinoma, brain cancer, thyroid cancer, and other cell lines (Fig. 1C). Tumor cell line analysis showed widespread expression of RNF216 in LIHC 1576, U2OS, ACHN, and others (Fig. 1D). Along with these findings, our assessment of 5 GEO datasets revealed that RNF216 is overexpressed in LIHC, COAD, and LUAD (Fig. 1E-I). For explicitly verifying the protein expression pattern of RNF216, we collected IHC staining images from the HPA database, which demonstrated increased RNF216 expression levels in COAD and LUAD (Fig. 1J-K).

### Diagnostic value of RNF216 in pan-cancer

**Figure 2:**
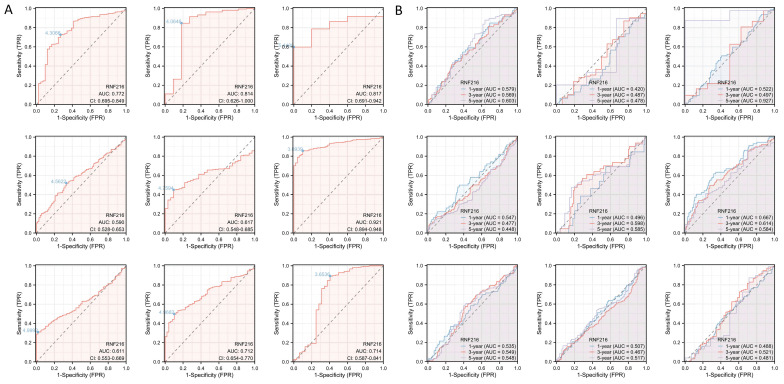
** Diagnostic value of RNF216 across pan-cancer.** (A) ROC curves for the diagnosis of RNF216. (B) Time-dependent ROC curves for RNF216.

RNF216 demonstrates strong diagnostic value across multiple cancers, including COAD (AUC=0.772, 95% confidence interval (CI): 0.695-0.849), ESCA (AUC=0.814, 95% CI: 0.626-1.000), GBM (AUC=0.817, 95% CI: 0.691-0.942), KIRC (AUC=0.590, 95% CI: 0.528-0.653), KIRP (AUC=0.617, 95% CI: 0.548-0.685), LIHC (AUC=0.921, 95% CI: 0.894-0.948), LUAD (AUC=0.611, 95% CI: 0.553-0.669), LUSC (AUC=0.712, 95% CI: 0.654-0.770), and STAD (AUC=0.714, 95% CI: 0.587-0.841) (Fig. 2A). Additionally, time-series ROC analysis indicated that RNF216 has high predict value for 1-year, 3-year, and 5-year OS in COAD, ESCA, GBM, KIRP, LIHC, LUAD, LUSC, and STAD (Fig. 2B).

### Prognostic value of RNF216 in pan-cancer

**Figure 3:**
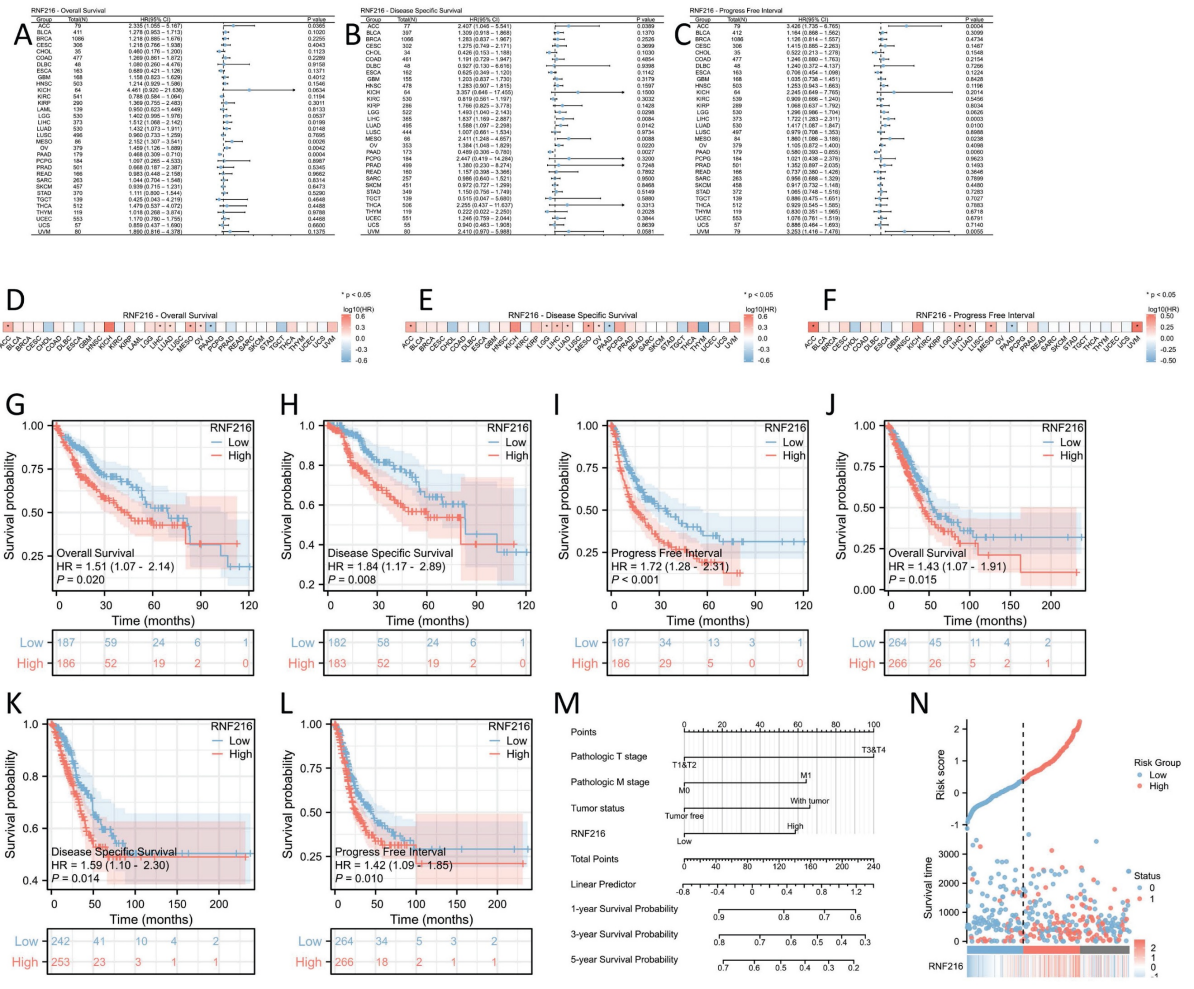
** Prognostic value of RNF216 across pan-cancer.** (A-C) Forest plots showing the results of univariate Cox regression analysis of RNF216 on OS, DSS, and PFI in TCGA pan-cancer. (D-F) Heatmaps illustrating the univariate Cox regression results for RNF216 on OS, DSS, and PFI in TCGA pan-cancer. (G-I) Correlations between RNF216 and OS, DSS, and PFI in LIHC. (J-L) Correlations between RNF216 and OS, DSS, and PFI in LUAD. (M) A prognostic nomogram highlighting the significance of RNF216 in LIHC. (N) Distribution of RNF216 expression levels and survival status: 0 indicates dead; 1 indicates alive.

To evaluate the prognostic value of RNF216, multivariate Cox regression analysis was performed to analyze its effect on OS, DSS, and PFI across different cancer types. Forest plots (Fig. 3A-C) and prognostic heatmaps (Fig. 3D-F) showed that RNF216 has prognostic relevance in multiple cancers. Survival analysis further indicated that high RNF216 expression is linked to poorer outcomes in LIHC and LUAD (Fig. 3G-L). Additionally, the prognostic importance of RNF216 was confirmed within specific LIHC and LUAD subgroups, showing consistent and significant associations. For example, in LIHC, age (hazard ratio (HR) = 1.96, 95% CI: 1.14-3.37, *P* = 0.015), BMI (HR = 1.8, 95% CI: 1.07-3.04, *P* = 0.027), and sex (HR = 1.79, 95% CI: 1.14-2.82, *P* = 0.012) all demonstrated significance (Fig. S2A). In LUAD, factors such as anatomic neoplasm subdivision (HR = 1.47, 95% CI: 1.01-2.15, *P* = 0.045), tumor location (HR=1.98, 95% CI: 1.11-3.52, *P* = 0.020), and pathological stage (HR=1.67, 95% CI: 1.03-2.71, *P* = 0.036) were also significant (Fig. S2B).

In Cox regression models, univariate analysis identified pathological T stage (*P* < 0.001), pathological M stage (*P* = 0.017), tumor status (*P* < 0.001), and RNF216 level (*P* = 0.020) as risk factors for LIHC-adjusted OS. Multivariate Cox regression presented that pathological T stage (*P* < 0.001), tumor status (*P* = 0.013), and RNF216 expression level (*P* = 0.028) were independent risk factors for OS in LIHC (Tab. 1). Based on these findings, we carried out a prognostic nomogram for LIHC that includes RNF216 expression levels (Fig. 3M). The risk score plot illustrates the correlation between RNF216 expression and survival outcomes in LIHC cases. The density of red dots representing deaths increased with higher risk scores, indicating that elevated RNF216 expression was significantly linked to an increased chance of death in LIHC cases (Fig. 3N).

### Relationship between RNF216 expression and clinicopathologic features in LIHC and LUAD

**Figure 4:**
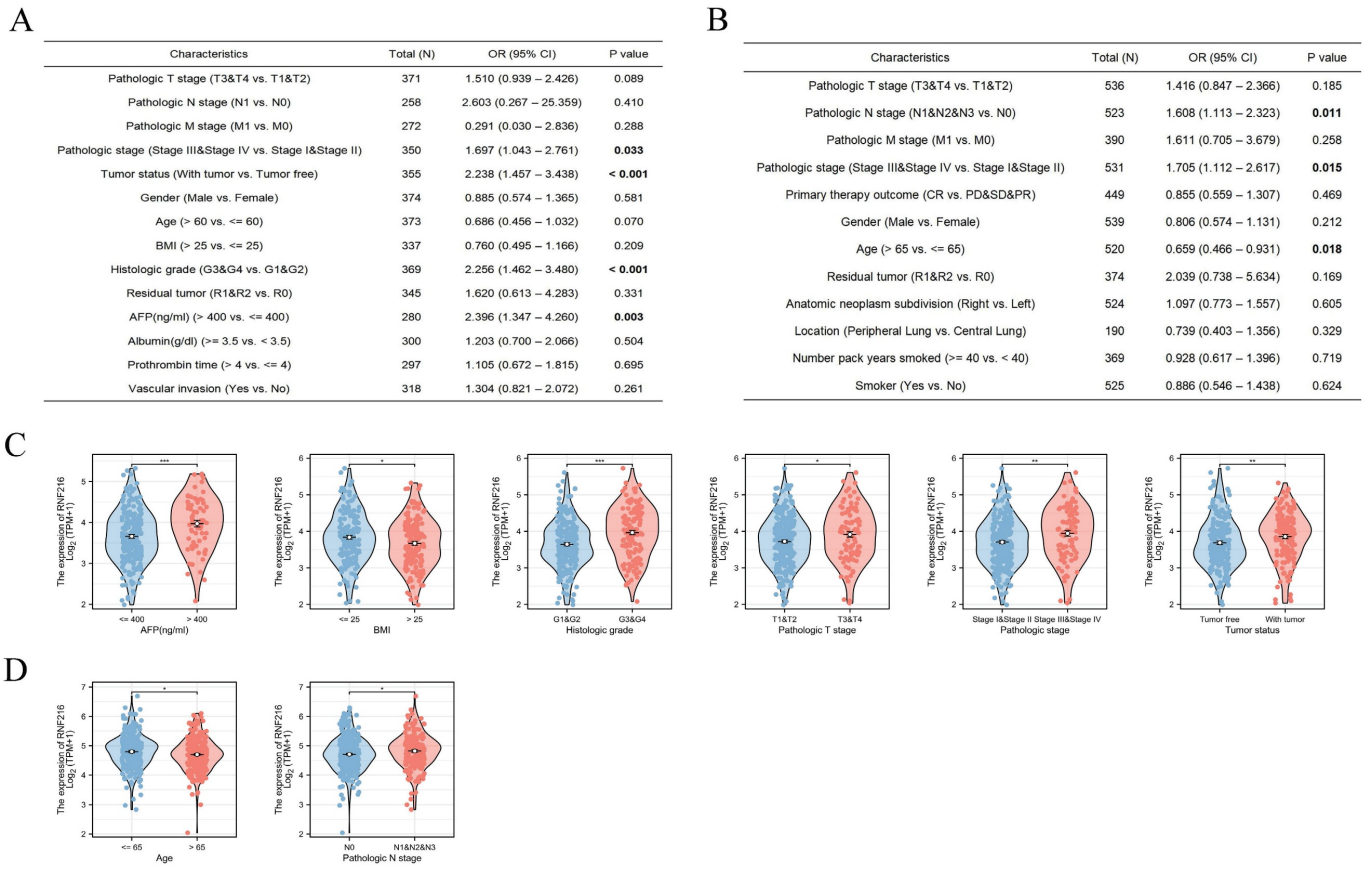
** Clinical significance of RNF216 in LIHC and LUAD.** Multivariate logistic analysis examining the relationship between RNF216 expression and other clinical indicators in LIHC (A) and LUAD (B). (C) The associations of RNF216 expression with AFP, BMI, histological grade, pathological T stage, pathological stage, and tumor status in LIHC. (D) The associations of RNF216 expression with age and pathological N stage in LUAD. (*P < 0.05; **P < 0.01; ***P < 0.001).

Using multivariate logistic regression analysis, we found that high RNF216 expression was linked to advanced pathological stage (odds ratio (OR) = 1.697, 95% CI: 1.043-2.761, *P* = 0.033), tumor-positive status (OR = 2.238, 95% CI: 1.457-3.438, *P* < 0.001), higher histological grade (OR = 2.256, 95% CI: 1.462-3.480, *P* < 0.001), and > 400 ng/mL AFP (OR = 2.396, 95% CI: 1.347-4.260,* P* = 0.003) in LIHC (Fig. 4A). Similarly, high RNF216 expression was bound up with advanced pathological N stage (OR = 1.608, 95% CI: 1.113-2.323, *P* = 0.011), advanced pathological stage (OR = 1.705, 95% CI: 1.112-2.617, *P* = 0.015), and age ≤ 65 years (OR = 0.659, 95% CI: 0.466-0.931, *P* = 0.018) in LUAD (Fig. 4B). In subgroup analyses, we observed that high expression of RNF216 was related to LIHC in cases with AFP > 400 ng/mL, BMI ≤ 25 kg/m², higher histological grade, advanced pathological T stage, advanced pathological N stage, and with tumor (Fig. 4C). Similarly, it was associated with age ≤ 65 years and advanced pathological N stage in LUAD patients (Fig. 4D).

### Mutations in the RNF216 gene in pan-cancer analyses

**Figure 5:**
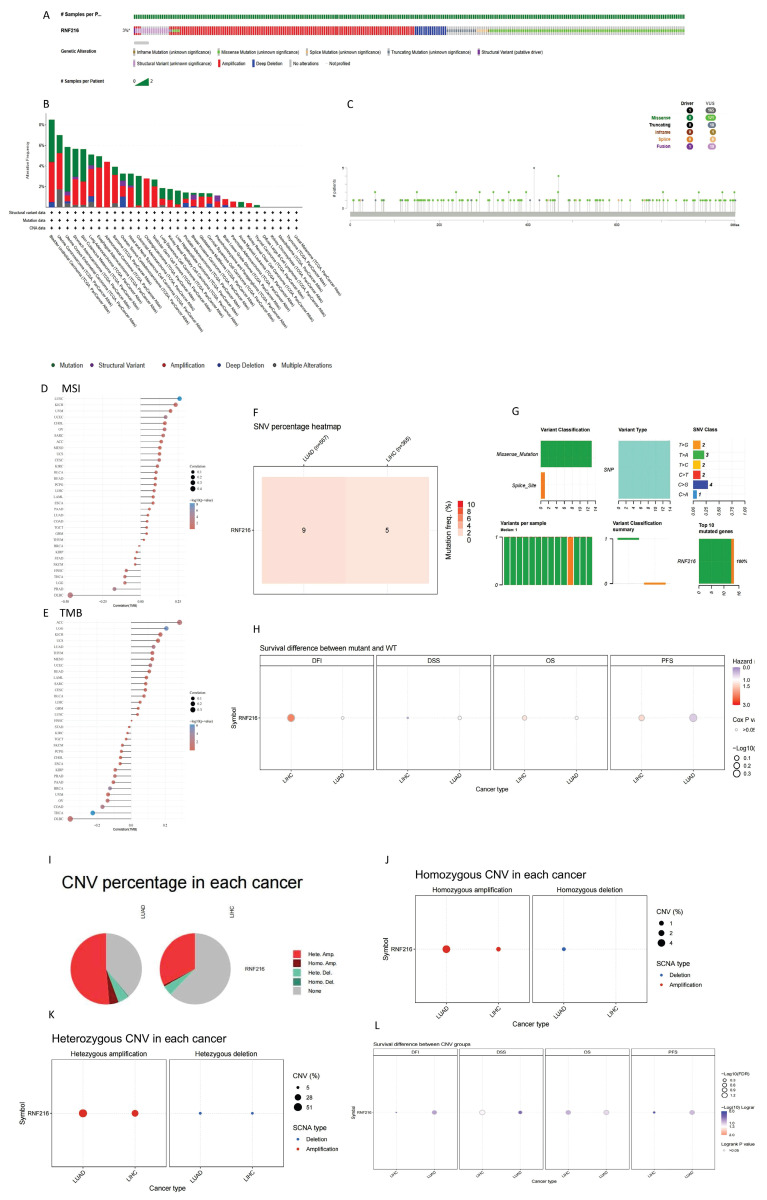
** Genetic mutation analysis of RNF216.** (A) Mutation status of RNF216 across pan-cancer. (B) Alteration frequency and mutation types of RNF216 in pan-cancer. (C) Visualization of RNF216 mutation sites in pan-cancer. (D, E) TMB and MSI correlation analyses for RNF216 in pan-cancer. (F) SNV percentage heatmap of RNF216 in LUAD and LIHC. (G) SNV classes of RNF216 in LUAD and LIHC. (H) Survival differences between the RNF216 mutation and wild type in LUAD and LIHC. (I) Pie chart of CNV percentages of RNF216 in LUAD and LIHC. (J) Homozygous CNV of RNF216 in LUAD and LIHC. (K) Heterozygous CNV of RNF216 in LUAD and LIHC. (L) Survival differences between CNV and wild type in LUAD and LIHC.

To evaluate mutations in RNF216 across pan-cancer, we implemented an overall analysis by the cBioPortal database and found that RNF216 mutated in 3% of pan-cancer cases (Fig. 5A). Additionally, our research of RNF216 alteration frequencies across different tumor types revealed the highest mutation rates in bladder urothelial carcinoma, uterine carcinoma, and endometrial carcinoma of the uterus. Significantly, amplification was verified as the most common mutation type of RNF216 (Fig. 5B). Detection of RNF216 mutation sites across pan-cancer identified 166 distinct sites spanning amino acids 0 to 866 (Fig. 5C). Furthermore, positive correlations between RNF216 expression and MSI were observed in 24 tumor types, while positive correlations with TMB were found in 16 tumor types, revealing that RNF216 notably influences both TMB and MSI (Fig. 5D, E). We also examined single-nucleotide variants (SNVs) in RNF216 across LIHC and LUAD, creating a heatmap of SNV frequencies in these cancers (Fig. 5F). These 14 SNVs included 13 missense mutations and 1 splice site mutation. We summarized SNV types (Fig. 5G) and performed survival analyses comparing mutant and wild-type RNF216 in LIHC and LUAD (Fig. 5H). Additionally, we analyzed copy number variation (CNV) of RNF216, which mainly shows heterozygous amplification in both LIHC and LUAD (Fig. 5I). The CNV landscape of RNF216 in LIHC and LUAD is presented from both homozygous (Fig. 5J) heterozygous (Fig. 5K) and perspectives (Fig. 5L).

### Differential expression genes and enrichment analysis in LIHC and LUAD

**Figure 6:**
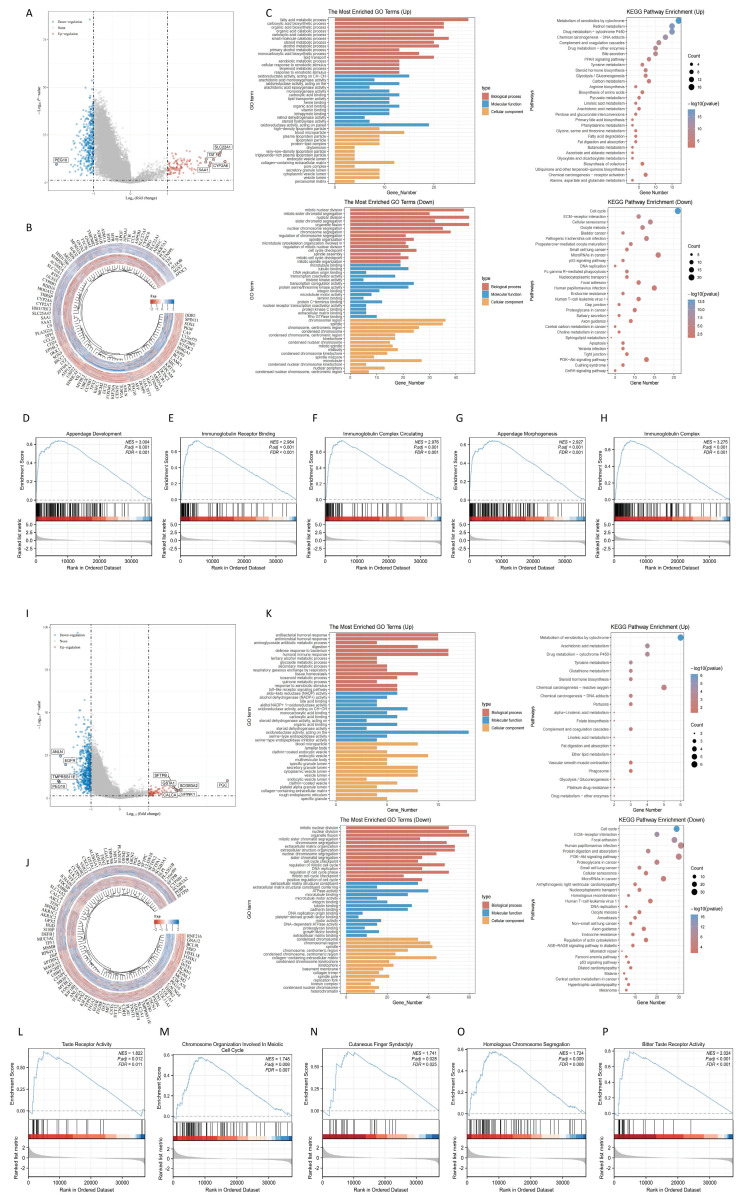
** Differentially expressed genes (DEGs) and enrichment analysis of RNF216 in LIHC and LUAD.** (A) Volcano plot of DEGs in LIHC. (B) Heatmap of DEGs in LIHC. (C) GO and KEGG enrichment analysis of RNF216 DEGs in LIHC. (D-H) GSEA analysis comparing the RNF216 low-expression group and the high-expression group, along with the correlation analysis of RNF216 with core genes in each pathway in LIHC. (I) Volcano plot of DEGs in LUAD. (J) Heatmap of DEGs in LUAD. (K) GO and KEGG enrichment analysis of RNF216 DEGs in LUAD. (L-P) GSEA analysis comparing the RNF216 low-expression and high-expression groups, along with the correlation analysis of RNF216 with core genes in each pathway in LUAD.

Through single-gene differential analysis of the RNF216-related genes, 464 DEGs were identified in LIHC (353 downregulated and 111 upregulated) (Fig. 6A, B). To investigate the functional implications of RNF216, GO and KEGG enrichment analyses were performed on the RNF216-associated DEGs. In LIHC, GO enrichment analysis indicated that upward-adjusted DEGs were significantly enriched in fatty acid metabolism, oxidoreductase activity, and blood microparticles, while downward-adjusted DEGs participated in mitosis, transcription coregulatory activity, and chromosomal regions. KEGG analysis revealed that upward-adjusted DEGs were notably funneled into the metabolism of xenobiotics by cytochrome, retinol metabolism, and drug metabolism-cytochrome P450, whereas downward-adjusted DEGs were associated with the cell cycle, microRNA in cancer, and human papillomavirus infection (Fig. 6C). Next, to identify RNF216-associated enriched pathways, we performed GSEA analysis. It showed that in LIHC, high RNF216 expression was linked to appendage development (NES = 3.004, *P*.adj < 0.001), immunoglobulin receptor binding (NES = 2.984, *P*.adj < 0.001), circulating immunoglobulin complex (NES = 2.976, *P*.adj < 0.001), appendage morphogenesis (NES = 2.927,* P*.adj < 0.001), and immunoglobulin complex assembly (NES = 3.275, *P*.adj < 0.001). (Fig. 6D-H).

In LUAD, 649 DEGs were found (570 downregulated and 79 upregulated) (Fig. 6I-J). In LUAD, GO enrichment analysis illustrated that upregulated DEGs were significantly funneled into bacterial defense response, oxidoreductase activity, and endosomal vesicles. Downregulated DEGs were enriched in organelle fission, ATPase activity, and the collagen-containing extracellular matrix. KEGG enrichment analysis showed that upregulated DEGs were notably involved in cytochrome-mediated xenobiotic metabolism, chemical carcinogenesis-reactive oxygen species, and arachidonic acid metabolism, while downregulated DEGs were linked to human papillomavirus infection, the PI3K-Akt signaling pathway, and the cell cycle (Fig. 6K). High RNF216 expression was associated with taste receptor activity (NES = 1.822, *P*.adj = 0.012), chromosome organization involved in the meiotic cell cycle (NES = 1.748, *P*.adj = 0.008), cutaneous finger syndactyly (NES = 1.741, *P*.adj = 0.028), homologous chromosome segregation (NES = 1.724, *P*.adj = 0.009), and bitter taste receptor activity (NES = 2.024, *P*.adj < 0.001). Correlation analysis showed that core factors from 10 pathways in these tumors correlated with RNF216 expression (Fig. 6L-P). These results suggest that RNF216 may influence downstream pathways by targeting these molecules, offering new therapeutic targets for future anticancer drugs.

### RNF216 co-expression genes and PPI network in LIHC and LUAD

**Figure 7:**
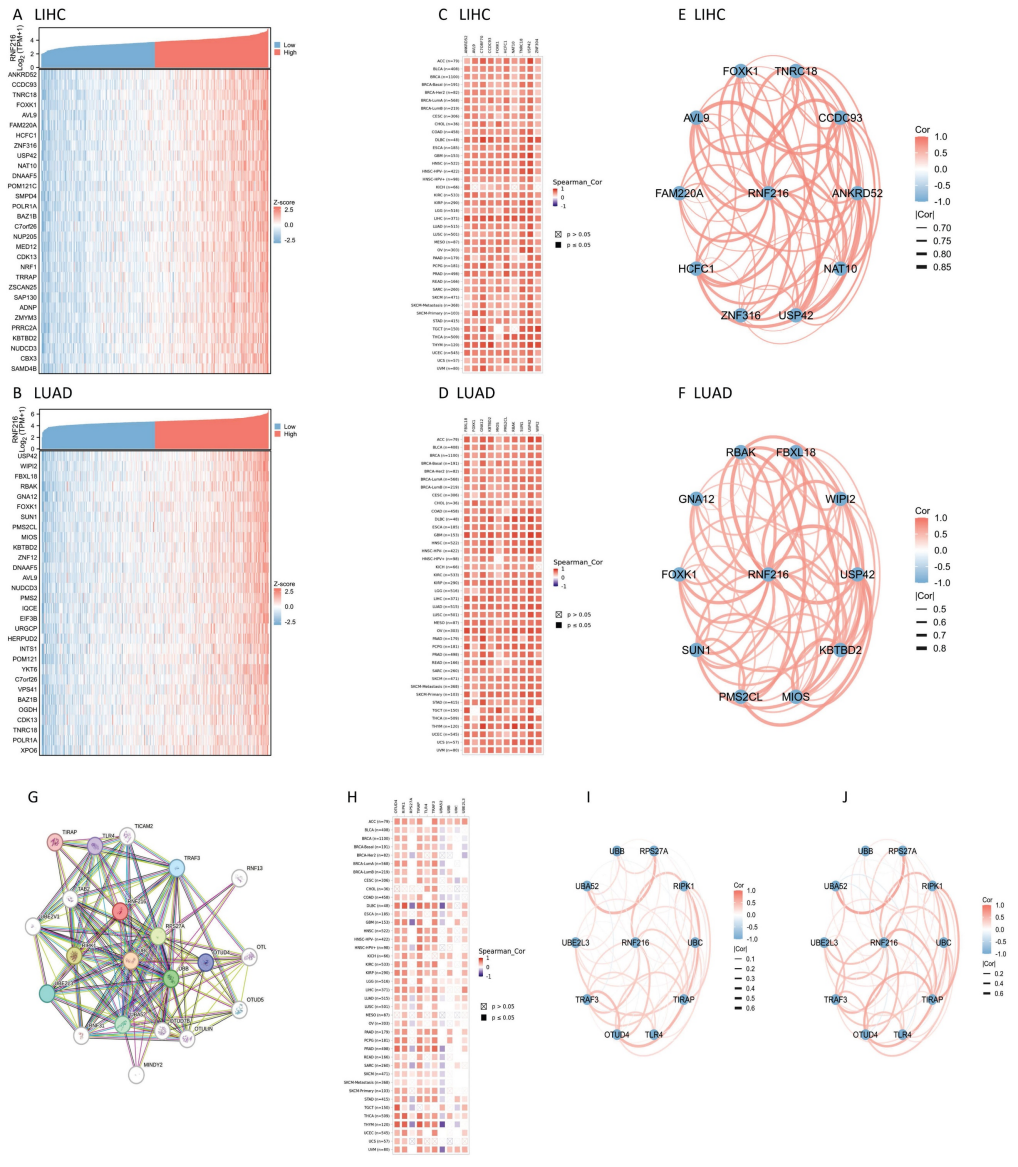
** RNF216-related genes and PPI network.** (A-B) Heatmap of the top 30 co-expressed genes of RNF216 in LIHC and LUAD. (C-D) Heatmap of the top 10 correlations in the co-expression network for LIHC and LUAD. (E-F) Correlation of each of the top 10 genes in LIHC and LUAD. (G) The top 20 RNF216-related proteins identified through PPI network analysis. (H) Heatmap of the top 10 correlations in the PPI network across pan-cancer. (I-J) Correlation of each of the top 10 genes in LIHC and LUAD.

We examined co-expressed genes of RNF216 in LIHC and LUAD by utilizing RNA sequencing data from the TCGA database, visualizing 30 genes with the strongest correlation (Fig. 7A, B). Next, we assessed the expression patterns of the top 10 genes with the strongest correlation across pan-cancer, where most showed positive correlations with RNF216 (Fig. 7C, D) and exhibited significant correlations among themselves (Fig. 7E, F). By the STRING tool, we verified 20 proteins interacting with RNF216 (Fig. 7G). Additionally, we studied the co-expression and gene correlations of the top 10 genes with the highest interaction points across pan-cancer (Fig. 7H). Results consistently presented that these genes were co-expressed in the majority of tumors and had strong correlations among each other in LIHC and LUAD (Fig. 7I, J).

### Prognostic significance of RNF216 co-expressed genes in LIHC and LUAD

**Figure 8:**
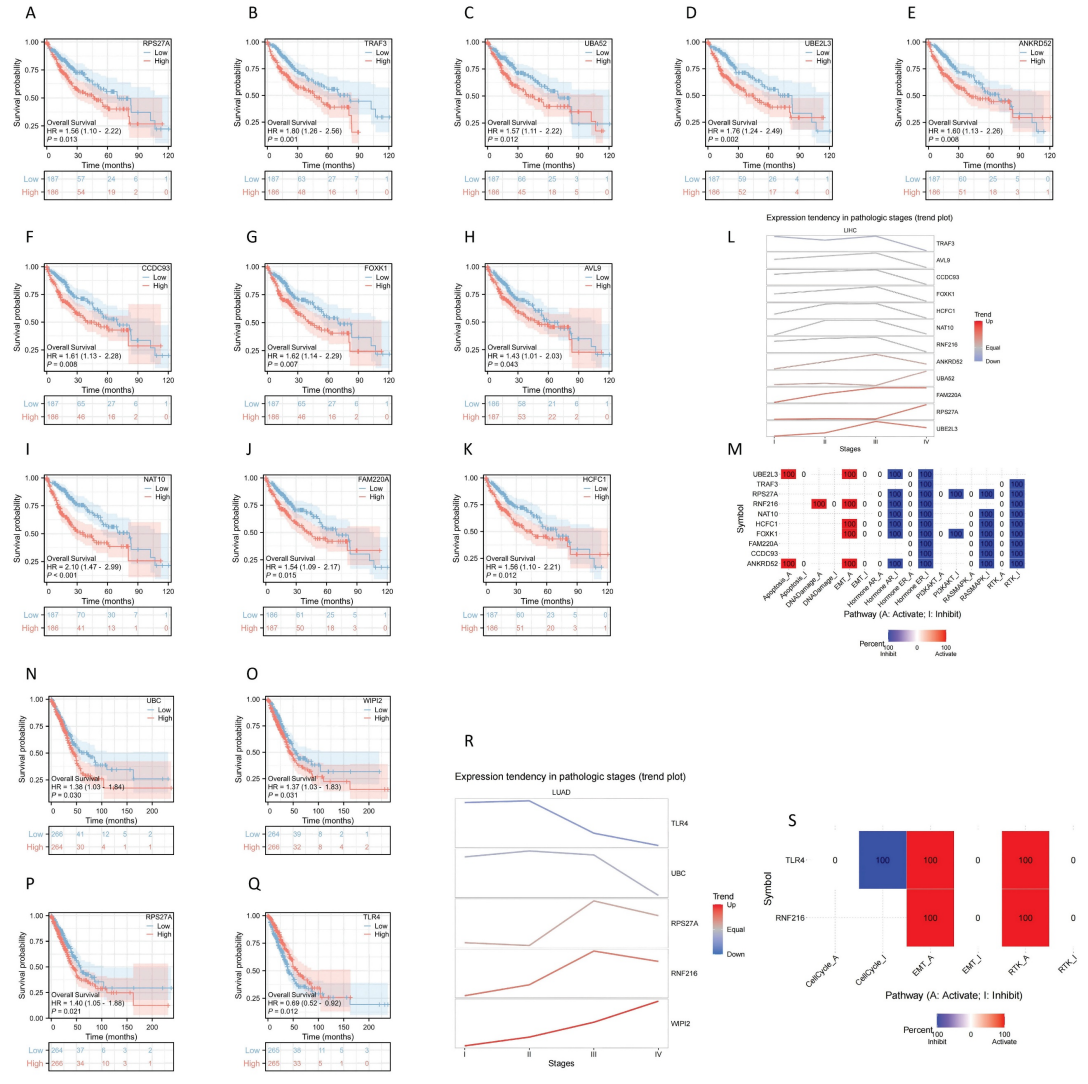
**Prognostic value analysis of RNF216 and co-expression genes.** (A-K) Prognostic significance of RPS27A, TRAF3, UBA52, UBE2L3, ANKRD52, CCDC93, FOXK1, AVL9, NAT10, FAM220A, and HCFC1 in LIHC. (L) Expression patterns of RNF216-related genes across different pathological stages of LIHC. (M) Potential common functional pathway of RNF216-related genes in LIHC. (N-Q) Prognostic significance of UBC, WIPI2, RPS27A, and TLR4 in LUAD. (R) Expression patterns of RNF216-related genes across different pathological stages of LUAD. (S) Potential common functional pathway of RNF216-related genes in LUAD.

Analysis of the prognostic significance of the previously mentioned genes revealed that 11 genes had significance in LIHC: RPS27A (HR = 1.56, 95% CI: 1.10-2.22,* P* = 0.013), TRAK3 (HR = 1.80, 95% CI: 1.26-2.56, *P* = 0.001), UBA52 (HR = 1.57, 95% CI: 1.11-2.22, *P* = 0.012), UBE2L3 (HR = 1.76, 95% CI: 1.24-2.49, *P* = 0.002), ANKRD52 (HR = 1.60, 95% CI: 1.13-2.26, *P* = 0.008), CCDC93 (HR = 1.61, 95% CI: 1.13-2.28, *P* = 0.008), FOXK1 (HR = 1.62, 95% CI: 1.14-2.29, *P* = 0.007), AVL9 (HR = 1.43, 95% CI: 1.01-2.03, *P* = 0.043), NAT10 (HR = 2.10, 95% CI: 1.47-2.99, *P* < 0.001), FAM220A (HR = 1.54, 95% CI: 1.09-2.17,* P* = 0.015), HCFC1 (HR = 1.56, 95% CI: 1.10-2.21, *P* = 0.012) (Fig. 8A-K). We further analyzed gene expression across LIHC tumor stages, showing that expression of ANKRD52, UBA52, FAM220A, RPS27A, and UBE2L3 increased progressively with LIHC progression (Fig. 8L). Using GSCALite, we evaluated the potential roles of RNF216 and these 11 genes in LIHC. The analysis hints that these genes may contribute to LIHC development by regulating mechanisms such as epithelial-mesenchymal transition (EMT) and Hormone AR (Fig. 8M).

Four genes showed prognostic significance in LUAD: UBC (HR = 1.38, 95% CI: 1.03-1.84,* P* = 0.030), WIPI2 (HR = 1.37, 95% CI: 1.03-1.83, *P* = 0.031), RPS27A (HR = 1.56, 95% CI: 1.10-2.22, *P* = 0.013), and TLR4 (HR = 0.69, 95% CI: 0.52-0.92, *P* = 0.012) (Fig. 8N-Q). We further analyzed gene expression across LUAD tumor stages, showing that expression of RPS27A and WIPI2 levels increased with LUAD advancement (Fig. 8R). Using GSCALite, we evaluated the potential effects of RNF216 and these 4 genes in LUAD. The analysis hints that these genes may contribute to LUAD development by regulating mechanisms such as cell cycle, epithelial-mesenchymal transition (EMT), and RTK (Fig. 8S).

### Correlation between RNF216 expression and TIME in pan-cancer

**Figure 9:**
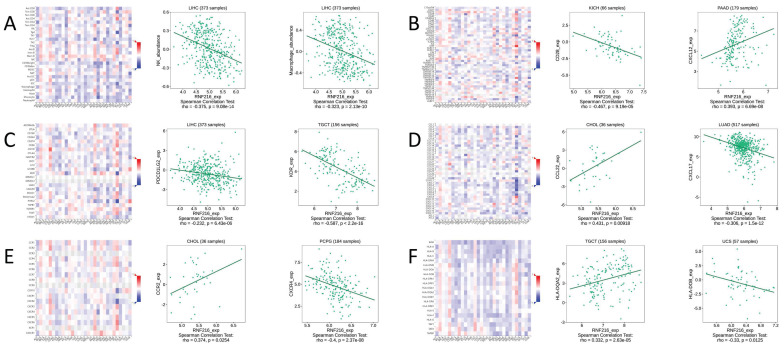
** Correlations of RNF216 expression with immunomodulators.** A-F display the correlations between RNF216 expression and (A) lymphocytes, (B) immune stimulators, (C) immune inhibitors, (D) MHC molecules, (E) chemokines, and (F) receptors in the TISIDB database. Red and blue colors indicate positive and negative correlations, respectively.

We conducted gene co-expression analysis using the TISIDB database to evaluate the correlation between RNF216 expression level and different TIME components. We found obvious relationships between RNF216 expression and a variety of immunologic factors across different cancers. Specially, RNF216 expression was negatively related to NK cell and macrophage subpopulations in LIHC (Fig. 9A). Among the 45 immune stimulators examined, RNF216 expression showed a negative correlation with CD28 in kidney interstitial cell carcinoma (KICH) and a positive correlation with CXCL12 in pancreatic adenocarcinoma (PAAD) (Fig. 9B). Analysis of 24 immunosuppressive factors revealed negative correlations between RNF216 expression and PDCD1LG2 in LIHC, and between RNF216 expression and KDR in tenosynovial giant cell tumor (TGCT) (Fig. 9C). Fig. 9D shows that RNF216 positively relates to CCL22 in CHOL and negatively relates to CXCL17 in LUAD. Additionally, our chemokine analysis identified a positive relationship between RNF216 and CCR2 in CHOL, and a negative relationship with CXCR4 in pheochromocytoma and paraganglioma (PCPG) (Fig. 9E). Receptor analysis demonstrated positive correlations between RNF216 and HLA-DQA2 in TGCT, and negative correlations with HLA-DOB in uterine carcinosarcoma (UCS) (Fig. 9F). Overall, these findings underscore RNF216's broad potential to predict pan-cancer immune-related phenotypes.

### Single-cell expression of RNF216 in LIHC

**Figure 10:**
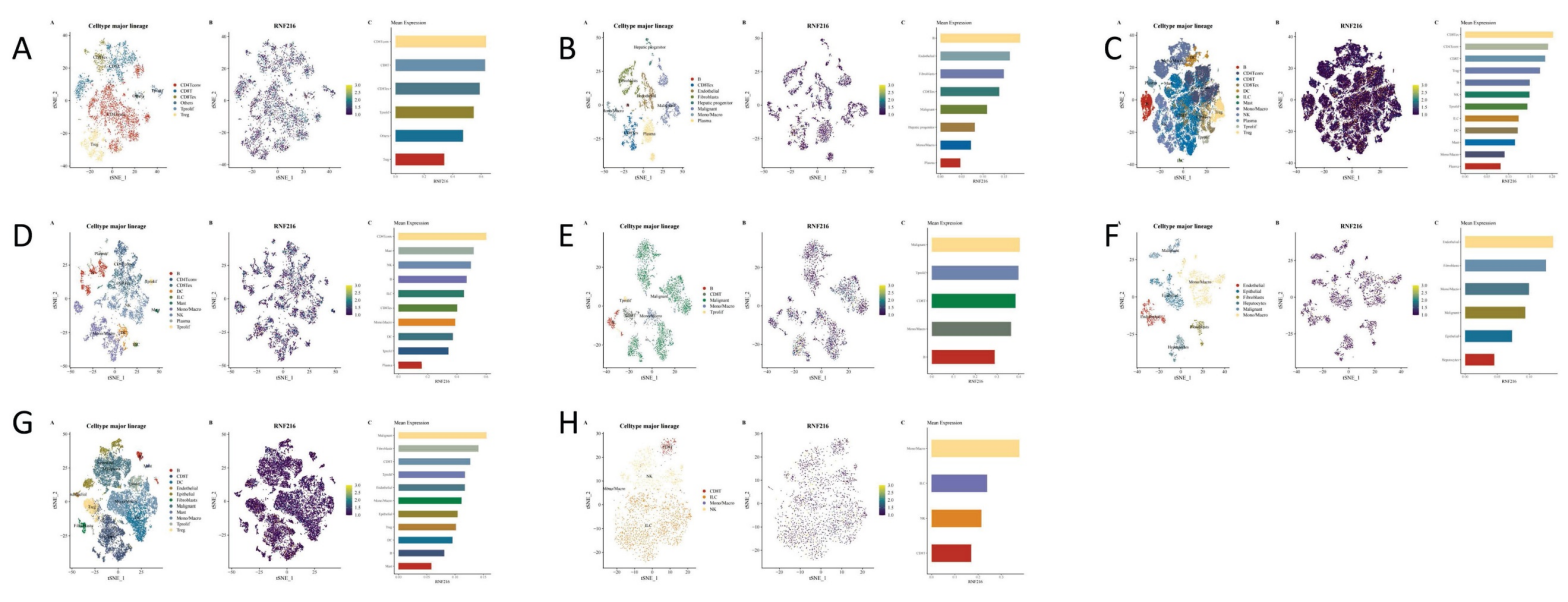
** The RNF216 expression analysis at the single-cell level in LUAD and LIHC across GSE98638.** (A) GSE125449 (B) GSE140228_10x (C) GSE140228_Smartseq2 (D) GSE146115 (E) GSE146409 (F) GSE166635 (G) and GSE179795 (H).

We studied the correlation between immune cells and RNF216 expression using data from eight separate LIHC datasets in the TISCH2 database. Clustering plots revealed that RNF216 expression patterns differ across immune cell types, emphasizing its importance to the LIHC immune environment. In the GSE98638 and GSE140228_Smart-seq2 datasets, RNF216 showed the highest expression levels in the CD4Tconv cell cluster; in the GSE146115 and GSE166635 datasets, it was most highly expressed in the malignant cell cluster. Additionally, peaks in RNF216 expression were observed in monocyte/macrophage (Mono/Macro) (GSE179795), B cells (GSE125449), CD8 Tex (GSE140228_10x), and endothelial cells (GSE146409) (Fig. 10A-H).

### Analysis of immunotherapy and chemotherapy efficacy in RNF216

**Figure 11:**
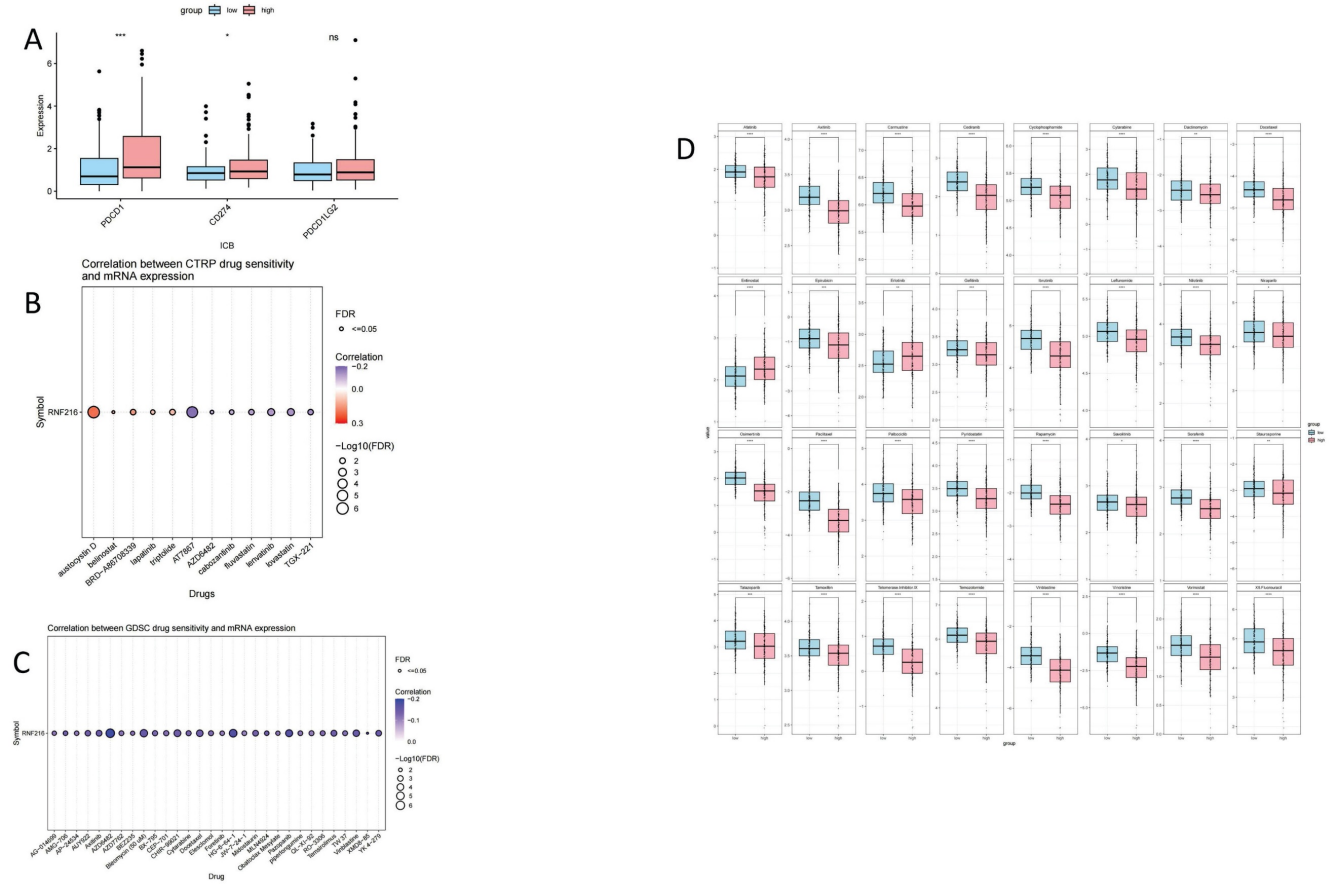
** The relationship between the expression of RNF216 and responses to immunotherapy and chemotherapy.** (A) ICI-related markers in the RNF216-high and RNF216-low groups. (B) Correlation of CTRP drug sensitivity with RNF216 mRNA expression. (C) Correlation of GDSC drug sensitivity with RNF216 mRNA expression. (D) Relationship between RNF216 and chemotherapy drug sensitivity in LIHC. (*P < 0.05; **P < 0.01; ***P < 0.001).

To assess the clinical significance of RNF216 in immunotherapy, we compared ICI responses between samples with different expression levels of RNF216. ICI-related markers PD-1/PDCD1 and PD-L1/CD274 showed significantly lower PD-L1 TIDE scores in the RNF216-low expression group (Fig. 11A). Analysis of the CTRP and GDSC databases indicated that RNF216 expression levels were linked to sensitivity to several chemotherapeutic agents (such as Aflatoxin D, AT7867, AZD6482, and Protanib) across different cancer types (Fig. 11B-C). Further LIHC studies demonstrated lower IC50 values for afatinib, axitinib, carmustine, crizotinib, and cyclophosphamide in the high-RNF216 group, suggesting increased drug sensitivity in these tumors. In contrast, the low-expression group presented lower IC50 points for entacapone and erlotinib, leading to subtly increased drug sensitivity when RNF216 is downregulated (Fig. 11D).

### Correlation between RNF216 and genes related to ferroptosis and m6A methylation in LIHC and LUAD

**Figure 12:**
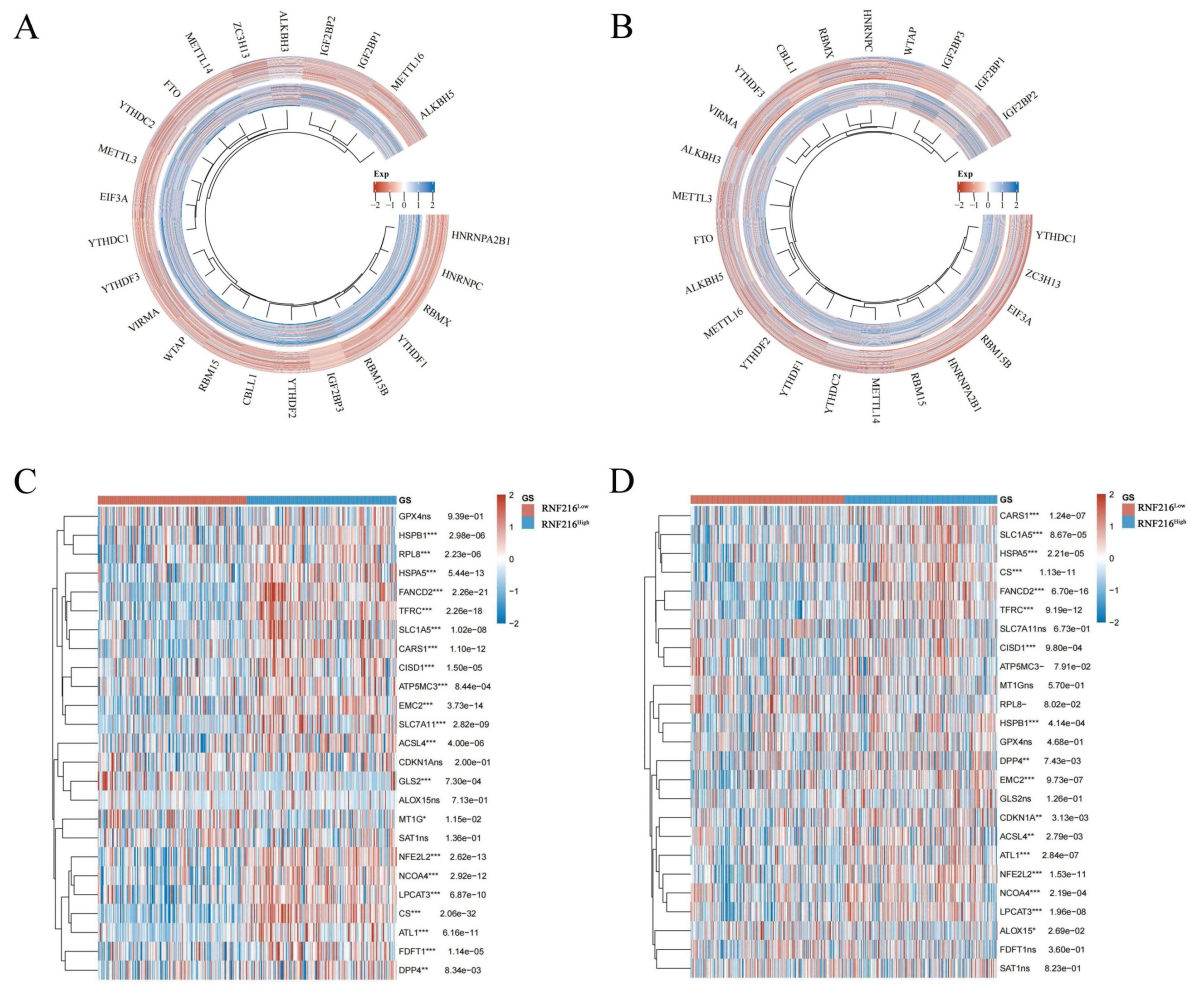
** Correlation of RNF216 with genes related to ferroptosis and m6A methylation in LIHC and LUAD.** (A, B) Correlation of RNF216 with these genes in LIHC. (C, D) Correlation of RNF216 with these genes in LUAD.

We assessed the relationship between RNF216 and genes related to ferroptosis and m6A methylation in LIHC and LUAD. Regarding m6A methylation, RNF216 expression was correlated with all m6A methylation-related genes in these two tumors, including METTL3, METTL14, WTAP, VIRMA, RBM15, RBM15B, ZC3H13, METTL16, CBLL1, YTHDC1, YTHDC2, YTHDF3, YTHDF1, YTHDF2, HNRNPC, IGF2BP1, IGF2BP2, IGF2BP3, RBMX, EIF3A, HNRNPA2B1, FTO, ALKBH5, and ALKBH3 (Fig. 12A, B). Concerning ferroptosis, RNF216 showed correlations with most genes in both tumor types. Specifically, in LIHC, HSPB1, RPL8, HSPA5, FANCD2, and TFRC were significantly linked to RNF216, while in LUAD, CARS1, SLC1A5, HSPA5, CS, and FANCD2 showed similar associations (Fig. 12C, D).

### The differential expression of RNF216 in clinical samples and LIHC cell lines

**Figure 13:**
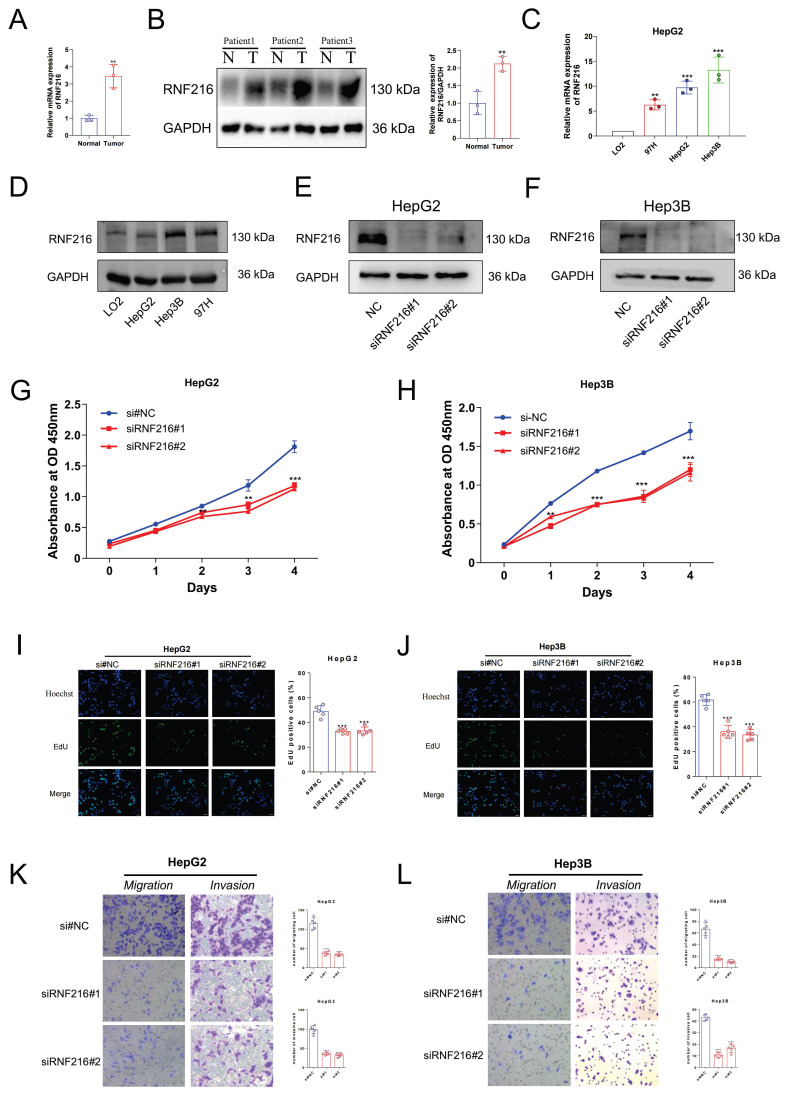
**RNF216 enhances the proliferation, migration, and invasion of LIHC cells *in vitro*.** (A, B) qRT-PCR analysis and Western Blot of RNF216 expression in tumor tissues. (C, D) qRT-PCR and WB analyses of RNF216 expression in LIHC cell lines. (E, F) Verification of RNF216-knockdown efficiency in HepG2 and Hep3B cells by immunoblotting. (G-J) RNF216 knockdown inhibited the proliferation of both HepG2 and Hep3B cells, as determined by CCK-8 assays (G, H) and EdU assays (I, J). Scale bar = 50 μm. (K, L) Knockdown of RNF216 inhibited migration (Transwell assays) and invasion (Matrigel invasion assays) of HepG2 and Hep3B cells. Student′s t-test was used for B, one-way ANOVA with Dunnett's post-hoc test was used for H-G. *P < 0.05, **P < 0.01, ***P < 0.001 and ****P < 0.0001.

We first detected the RNF216 expression in three pairs of freshly frozen tissues via quantitative real-time polymerase chain reaction (qRT-PCR) and Western Blot (WB). The qRT-PCR result showed (Fig. 13A) that the relative mRNA expression level of RNF216 in LIHC tissues was ~ 4-fold that in normal tissues. The WB results (Fig. 13B) demonstrated that the RNF216 protein expression level was notably increased in LIHC tissues in contrast to those in the corresponding normal tissues (fold change = 2.1, *P* < 0.01). The mRNA and protein expression levels of RNF216 in LIHC cell lines were checked by qRT-PCR and immunoblotting, respectively. Results revealed that RNF216 mRNA and protein expression levels were highly enhanced in HepG2, Hep3B, and MHCC97H cells, whereas its expression was significantly lower in LO2 cells (Fig. 13C, D). These findings indicated that RNF216 is abnormally upregulated in LIHC cells, suggesting a potential role in LIHC progression.

### Knockdown of RNF216 inhibits the proliferation, migration, and invasion of LIHC cells

To explore its functional significance, we knocked down its expression in HepG2 and Hep3B cells by small interfering RNAs (siRNAs). Both siRNAs significantly decreased the expression of RNF216 (Fig. 13E, F). Subsequently, CCK-8 and EdU assays were performed to evaluate the function of RNF216 knockdown on HepG2 and Hep3B cell proliferation. Results demonstrated that knockdown of RNF216 prohibited the proliferation of both cell lines (Fig. 13G-J). Quantitative analysis showed that the RNF216 knockdown reduced the proliferation rate of LIHC cells by approximately 30-50%, confirming that RNF216 is essential for maintaining the proliferative capacity of LIHC cells.

Transwell migration and invasion assays were carried out to investigate the function of RNF216 in LIHC cell motility. For the migration assay. The quantity of cells that migrated to the lower surface of the membrane was significantly lower in the siRNF216 groups than in the si-NC group (Fig. 13K, L). For the invasion assay, similar results were observed: RNF216 knockdown notably reduced the number of invasive HepG2 and Hep3B cells (Fig. 13K, L). Quantitative counting revealed that in contrast to the si-NC group, the migratory and invasive abilities of LIHC cells were reduced by approximately 50-70% after RNF216 knockdown (Fig. 13K, L). These data collectively indicated that RNF216 increases the migration and invasion of LIHC cells, which are critical processes for tumor metastasis.

## Discussion

This study aims to clarify the function of RNF216 in pan-cancer using bioinformatics and experimental techniques. First, we evaluated the expression of RNF216 across different human organs and tissues. Next, we analyzed the mRNA and protein levels of RNF216 between tumor and normal tissues. In addition, we assessed the prognostic and diagnostic value of RNF216 across different tumor types and analyzed its correlation with clinical data. We also investigated patterns of RNF216 mutations. Then, we examined differentially expressed and co-expressed genes of RNF216 in LIHC and LUAD, performed pathway enrichment analysis, and constructed PPI and GSEA networks to evaluate its prognostic value. Moreover, we analyzed RNF216's immune infiltration across pan-cancer types and its interaction with TIME. Finally, we evaluated the correlation between RNF216 expression and sensitivity to immunotherapy and chemotherapy, along with its association with ferroptosis and m6A methylation-related gene expression. To verify these findings, we carried out WB with clinical samples of LIHC. We selected three pairs of LIHC and corresponding normal tissues, and the figures illustrated notably increased expression of RNF216 in all three cancer tissues. We compared the RNF216 expression in a normal hepatocyte cell line and three LIHC cell lines, then transfected siRNA into two LIHC cell lines to knock down RNF216's expression. It validated that knocking down RNF216 decreased cell proliferation, migration, and invasion. This comprehensive research enhances our understanding of RNF216 as a pan-cancer therapeutic target, especially for LIHC and LUAD.

Ubiquitination is a post-translational modification (PTM) of proteins achieved through the conjugation of one or more ubiquitin molecules. This process involves the sequential action of E1 activators, E2 conjugases, and E3 ubiquitin ligases. In the first step of ubiquitination, E1 catalyzes the ATP-dependent activation of ubiquitin and leads to formation of a thioester bond between C terminus of ubiquitin and the catalytic cysteine on the E1. Then, E2 carries ubiquitin moieties transiently with a thioester linkage. Lastly, E3-ligase performs the transfer of ubiquitin from an E2 ubiquitin-conjugating enzyme to the targeted protein 39-40. E3 ligases play a vital role in tumor development and progression by focusing the destruction of tumor promoters or suppressors in human cancers 41. As an E3 ubiquitin ligase, the mechanism of action of RNF216 also explains its widespread distribution. Investigation of multiple databases shows that RNF216 is broadly distributed in the human body, especially in the reproductive, digestive, circulatory, musculoskeletal, endocrine, and immune systems. This suggests that RNF216 participates in various physiological functions and disease developments, such as testicular meiosis and spermatogenesis 42, autophagy 43, and bipolar disorder 44. Further pan-cancer analysis indicates that RNF216 expression varies significantly from normal tissues across most cancer types. The gene RNF216 and its product demonstrate clear diagnostic value in cancer, particularly in ESCA, GBM, and LIHC, where AUC values exceed 0.80. Combined with time-series ROC analysis, this shows that RNF216 has good sensitivity and specificity for tumor prediction. Forest plots and prognostic heatmaps reveal that higher levels of RNF216 are correlated with poorer OS, DSS, and PFI in LIHC, LUAD, and MESO. Interestingly, higher RNF216 levels are linked to better OS, DSS, and PFI in PAAD, which warrants further investigation into its underlying mechanisms. Among the four cancer types studied, LIHC and LUAD were selected for additional analysis. In most subgroups, RNF216 demonstrated strong predictive power for OS, corroborating the findings of Hao Ding *et al.* 45. Further analysis confirmed the clinical relevance of RNF216 in both cancer types.

RNF216 and its co-expressed genes, along with DEGs, demonstrate significant prognostic value across various cancers. Mutations in RNF216 may be connected to tumor initiation, progression, and outcomes. Several studies 46-47 have reported that RNF216 mutations are linked to Gordon-Homes syndrome. Our data indicate that alterations in RNF216 occur in 3% of pan-cancer patients, with amplification being the most common mutation type. MSI and TMB serve as novel biomarkers for recognizing cases who are apt to profit from immune checkpoint inhibitors 48. Consistent with these findings, in LIHC and LUAD, RNF216 expression shows significant positive correlations with both MSI and TMB levels. These results suggest that higher RNF216 expression may predict better survival outcomes following immunotherapy in LIHC patients. CNVs and SNVs are critical in complex diseases, and combining SNVs and CNVs studies can improve statistical power 49. For example, genes in the Neddylation pathway mostly show SNVs as missense mutations and CNVs as either heterozygous deletions or amplifications across various cancers, which are associated with poor prognosis 50. Our genetic analysis indicated high frequencies of SNVs and CNVs in LIHC and LUAD, with SNVs occurring at a rate of 1.5% (14/932) across both cancers. CNVs mostly appeared as heterozygous amplifications, which may drive gene expression changes that promote tumor growth.

To further explore interactions between RNF216 and other genes or proteins across different cancers, we analyzed RNF216-differentially expressed and co-expressed genes, performing GSEA and PPI analyses. Results showed that genes differentially expressed with RNF216 overexpression were involved in pathways such as chromosome organization during meiosis and homologous chromosome separation. Previous studies have reported that combined mutations of BRCA2 and TP53 cause meiotic arrest, which synergistically promotes proliferation and inhibits differentiation, contributing to carcinogenesis 51. Ji-Min Li *et al.* demonstrated that disrupting the RPA-RNF20 interaction leads to chromosome mis-segregation, defects in homologous recombination, and genomic instability—hallmark features of cancer cells that make them fragile 52. We hypothesize that RNF216 may promote LIHC and LUAD development by combining with these pathway genes and regulating related pathways. In the PPI network, hub genes showed positive correlations with RNF216 expression, indicating their participation in tumor biology. In these core genes, UBA52 promotes LIHC cell proliferation and migration by regulating EMC6 autophagy, while knockdown of UBA52 inhibits LIHC development and progression 53. TRAF6 enhances migration and invasion in LIHC cell lines via TLR4-induced NF-κB activation, whereas USP8 inhibits this process 54. Knockdown of USP22 may increase UBC expression, supporting pathways like cell cycle regulation and ubiquitin-mediated proteolysis during LUAD development 55. RPS27A promotes MDM2-mediated p53 ubiquitination and degradation, correlating with LUAD progression and poor prognosis 56. RIPK1 interacts with OTUD6B in LUAD cells, reducing ubiquitination levels, stabilizing proteins, and driving malignant progression 57. Additionally, using the GSCALite tool, we identified RNF216 and its ten core genes as potential drivers of LIHC progression through mechanisms such as cell death, DNA damage, EMT, and signal transduction. Notably, EMT shows a strong positive correlation with these hub genes. Cells undergoing EMT tend to adopt a more motile and invasive mesenchymal phenotype, allowing them to detach from the basement membrane and invade neighboring tissues—an essential step in cancer metastasis 58. This finding opens a pathway for future research into the interactions between RNF216 and other genes and their products across various cancers.

RNF216 is linked to immune infiltration and the immune microenvironment, influencing cancer prognosis by affecting the TIME. Using the TISIDB database, we found a negative relationship between RNF216 expression and that of NK cells and macrophages. Macrophages impact the TIME, while the tumor microenvironment has multiple effects on inflammation and tumor development. NK cells help fight tumors and promote inflammation by killing target cells and releasing cytokines 59. Experiments by Shahryar Khoshtinat Nikkhoi *et al.* 60, utilizing *in vivo* mouse tumor models, demonstrated that NK cells and macrophages are co-activated to carry out tumor-killing functions. Therefore, higher RNF216 expression may suppress the activity or presence of NK cells and macrophages. This suppression prevents tumor cells from immune responses, thus promoting tumor development and metastasis. In addition, our utilization of the TISCH2 database indicates that RNF216 is most abundant in CD4Tconv cell clusters and malignant cell clusters. This feature could guide strategies for immunotherapies such as immune checkpoint drugs, tumor vaccines, and cellular therapies 61, though further experimental validation is needed.

RNF216 is linked to immunotherapy, antitumor drug sensitivity, ferroptosis, and m6A methylation. In recent years, ICIs, especially PD-1 antibodies, have exhibited effectiveness in LIHC treatment comprehensively 62. TIDE can forecast patient reaction to immune checkpoint blockade by unpacking tumor gene expression profiles 63. We used the TIDE score to identify potential ICI-related biomarkers and discovered that decreased expression of RNF216 may relate to better ICI responses in LIHC. Additionally, we conducted a thorough analysis of the correlation between RNF216 expression and chemotherapy sensitivity by the GDSC and CTRP databases, focusing on its association with sensitivity to various chemotherapeutic drugs in LIHC. This analysis revealed a significant correlation with sensitivity to multiple anticancer agents. Notably, when RNF216 levels were low, the IC50 values for erlotinib and entegrastim decreased accordingly. Research by Tamara Zenz *et al.* 64 indicates that combining erlotinib with entuzumab effectively suppresses tumor growth in mice by promoting epidermal growth factor receptor inhibition in gastric cancer. This suggests that RNF216 could be a druggable target for developing tumor-specific chemotherapy or could assist in combination therapy by mediating interactions between different chemotherapeutic agents. Additionally, RNF216 expression correlates with ferroptosis- and m6A methylation-related gene expression in LIHC and LUAD, implying that higher RNF216 levels may be associated with poorer prognosis in these cancers. However, the underlying molecular mechanisms and clinical applications still require further experimental validation.

Several studies have indicated that inhibiting RNF216 can decrease the proliferation, invasion, and migration of tumor cells 11, 65. However, the impact of RNF216 on tumor biological behavior has not been fully confirmed through experiments. Considering the important role of RNF216 in LIHC observed in all our analyses, we carried out *in vitro* experiments by LIHC cell lines to further assess its role in tumor growth. After knocking out RNF216 in HepG2 and Hep3B cells, CCK-8 assays presented an obvious reduction in the cells' clonogenic ability in contrast to the control group, with the difference becoming more pronounced over time. Additionally, transwell experiments consistently showed a clear decrease in the migration and invasion capabilities of LIHC cells following RNF216 knockout, confirming that RNF216 promotes tumor growth in LIHC. These discoveries support the function of RNF216 in promoting cancer growth.

In this research, we carried out a preliminary inquiry into the function of RNF216 in LIHC and LUAD by collecting database data, clinical features, and experimental clues. The discoveries indicate that RNF216 expression in LIHC and LUAD is involved in tumor diagnosis and prognosis and could be important for immunotherapy and chemotherapy in LIHC. The novelty of our work lies in its extensive pan-cancer scope, which systematically establishes RNF216's potential as a multifaceted biomarker. More importantly, our findings highlight its clinical applicability, suggesting that RNF216 could not only serve as a prognostic indicator but also as a predictor for immunotherapy response and chemotherapy sensitivity in LIHC patients. The targeted inhibition of RNF216 emerges as a promising strategy to enhance anti-tumor efficacy 11, 17, 43, 65. While our study provides compelling evidence for the oncogenic role of RNF216 in LIHC and its significance across pan-cancer, several avenues for future investigation remain. Firstly, the pro-tumorigenic functions of RNF216 observed *in vitro* require validation in *in vivo* settings. We plan to employ xenograft mouse models with RNF216-knockdown LIHC cells to conclusively demonstrate its impact on tumor growth and metastasis. Secondly, the precise molecular mechanisms downstream of RNF216 need further elucidation. A critical next step is to identify its specific ubiquitination substrates, potentially through Co-IP/MS, to explain its regulation over processes like the cell cycle and EMT. Finally, the translational value of RNF216 as a therapeutic target warrants exploration. Future work will assess whether targeting RNF216, either alone or in combination with existing chemotherapy or immunotherapy, can yield synergistic anti-tumor effects *in vivo*. These planned studies will bridge the gap between our current findings and potential clinical applications, solidifying RNF216's position as a promising biomarker and therapeutic target in LIHC and other cancers.

## Supplementary Material

Supplementary figures.

## Figures and Tables

**Table 1:** Univariate and multivariate Cox regression analyses of clinicopathological features related to OS in LIHC.

Characteristics	Total(N)	Univariate analysis			Multivariate analysis	
		Hazard ratio (95% CI)	*P* value		Hazard ratio (95% CI)	*P* value
Pathologic T stage	370					
T1&T2	277	Reference			Reference	
T3&T4	93	2.598 (1.826 - 3.697)	**< 0.001**		2.486 (1.577 - 3.919)	**< 0.001**
Pathologic N stage	258					
N0	254	Reference				
N1	4	2.029 (0.497 - 8.281)	0.324			
Pathologic M stage	272					
M0	268	Reference			Reference	
M1	4	4.077 (1.281 - 12.973)	**0.017**		1.796 (0.412 - 7.824)	0.435
Tumor status	354					
Tumor free	202	Reference			Reference	
With tumor	152	2.317 (1.590 - 3.376)	**< 0.001**		1.828 (1.139 - 2.935)	**0.013**
Gender	373					
Female	121	Reference				
Male	252	0.793 (0.557 - 1.130)	0.200			
Age	373					
<= 60	177	Reference				
> 60	196	1.205 (0.850 - 1.708)	0.295			
BMI	336					
<= 25	177	Reference				
> 25	159	0.798 (0.550 - 1.158)	0.235			
Residual tumor	344					
R0	326	Reference				
R1&R2	18	1.604 (0.812 - 3.169)	0.174			
Histologic grade	368					
G1&G2	233	Reference				
G3&G4	135	1.091 (0.761 - 1.564)	0.636			
AFP(ng/ml)	279					
<= 400	215	Reference				
> 400	64	1.075 (0.658 - 1.759)	0.772			
Albumin(g/dl)	299					
< 3.5	69	Reference				
>= 3.5	230	0.897 (0.549 - 1.464)	0.662			
Prothrombin time	296					
<= 4	207	Reference				
> 4	89	1.335 (0.881 - 2.023)	0.174			
Child-Pugh grade	240					
A	218	Reference				
B&C	22	1.643 (0.811 - 3.330)	0.168			
Vascular invasion	317					
No	208	Reference				
Yes	109	1.344 (0.887 - 2.035)	0.163			
Adjacent hepatic tissue inflammation	236					
None	118	Reference				
Mild&Severe	118	1.194 (0.734 - 1.942)	0.475			
RNF216	373					
Low	187	Reference			Reference	
High	186	1.512 (1.068 - 2.142)	**0.020**		1.705 (1.058 - 2.747)	**0.028**
