# Supplementary material for: RNF216 as a Promising Biomarker for Prognosis, Immunotherapy, and Chemotherapy in LIHC: A Comprehensive Pan-Cancer Analysis and Experimental Validation
Source: J Cancer. 2026 Jan 30;17(3):483–506. doi: 10.7150/jca.125407 (PMC13003544; doi:10.7150/jca.125407)
Supplement: Supplementary file 1 — Supplementary figures. [file jcav17p0483s1.pdf]

Supplemental information

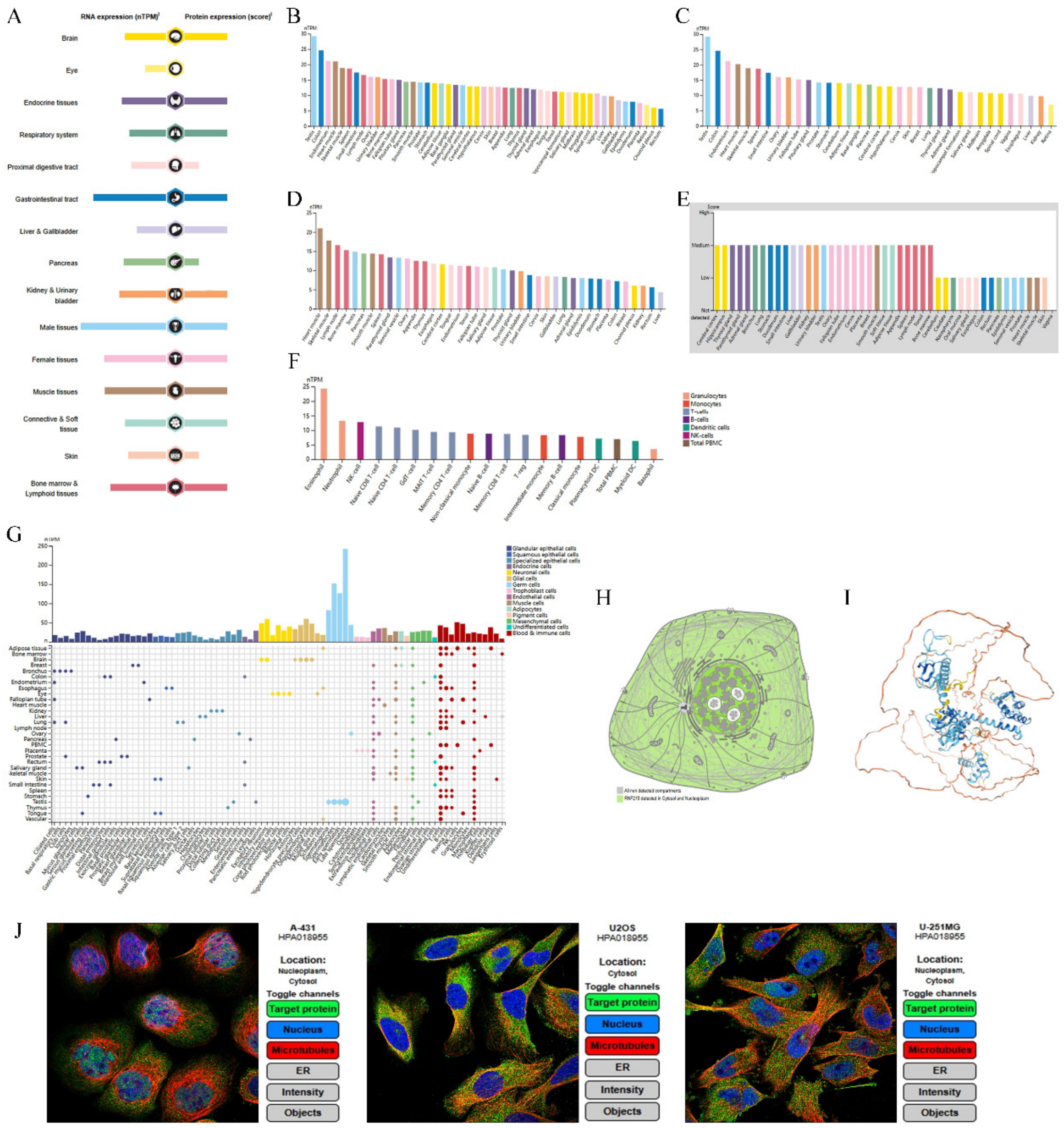

Figure S1. The expression of RNF216 in normal human organs and tissues. (A) Overview of RNF216 mRNA and protein expression across human organs and tissues. (B-D) RNF216 mRNA expression patterns across various tissues in the consensus, GTEx, and HPA databases. (E) RNF216 protein expression score across human organs in the HPA database. (F) RNF216 mRNA expression patterns across various immune cells in the HPA database. (G) Overview of RNF216 mRNA expression in multiple single-cell tissues. (H) Subcellular localization of RNF216. (I) The protein structure of RNF216. (J) The subcellular localization of RNF216, as depicted by immunofluorescence visualization in the HPA database.

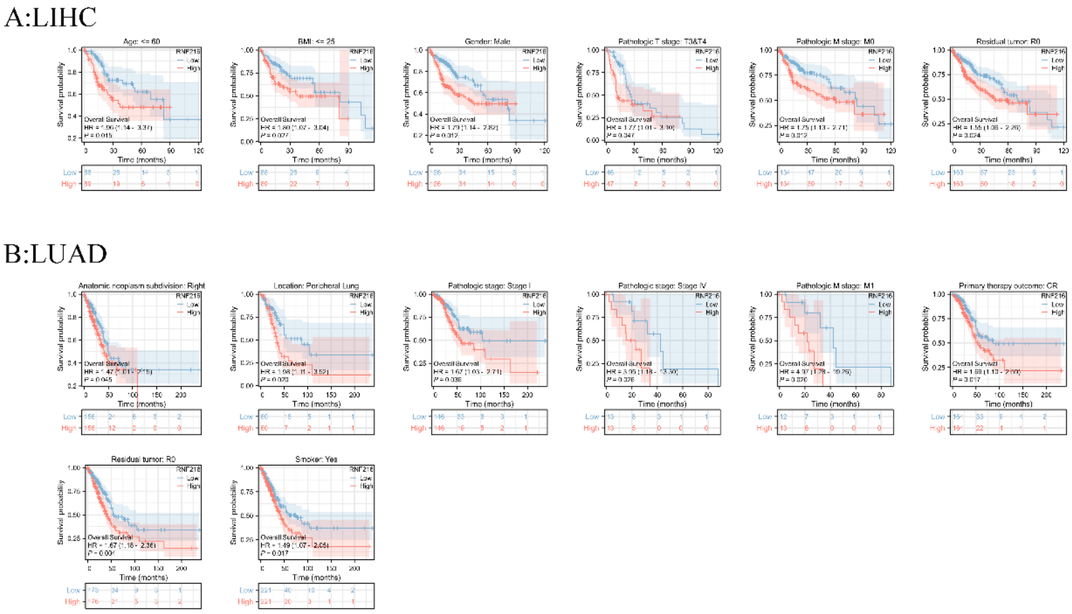

Figure S2. Prognostic value of RNF216 in LIHC and LUAD subgroups. (A) Prognostic significance of RNF216 in LIHC subgroups. (B) Prognostic significance of RNF216 in LUAD subgroups.
